# Evaluation of an unconditional cash transfer program targeting children’s first-1,000–days linear growth in rural Togo: A cluster-randomized controlled trial

**DOI:** 10.1371/journal.pmed.1003388

**Published:** 2020-11-17

**Authors:** Justine Briaux, Yves Martin-Prevel, Sophie Carles, Sonia Fortin, Yves Kameli, Laura Adubra, Andréa Renk, Yawavi Agboka, Magali Romedenne, Félicité Mukantambara, John Van Dyck, Joachim Boko, Renaud Becquet, Mathilde Savy

**Affiliations:** 1 NUTRIPASS, French National Research Institute for Sustainable Development–University of Montpellier-Montpellier SupAgro, Montpellier, France; 2 University of Bordeaux, Inserm, Institut de Recherche pour le Développement, UMR 1219, Bordeaux Population Health Research Center, Team IDLIC, Bordeaux, France; 3 Paris School of Economics, UMR 8545, Paris, France; 4 Projet de Développement Communautaire et Filets Sociaux, Ministère du Développement à la Base, Lomé, Togo; 5 UNICEF, Child Survival and Development, Lomé, Togo; 6 UNICEF, Social protection, Lomé, Togo; 7 The World Bank, Social Protection and Labor Global Practice, Washington, District of Columbia, United States of America; 8 The World Bank, Social Protection and Labor Global Practice, Country Office of Cotonou, Benin; London School of Hygiene and Tropical Medicine, UNITED KINGDOM

## Abstract

**Background:**

In 2014, the government of Togo implemented a pilot unconditional cash transfer (UCT) program in rural villages that aimed at improving children’s nutrition, health, and protection. It combined monthly UCTs (approximately US$8.40 /month) with a package of community activities (including behavior change communication [BCC] sessions, home visits, and integrated community case management of childhood illnesses and acute malnutrition [ICCM-Nut]) delivered to mother–child pairs during the first “1,000 days” of life. We primarily investigated program impact at population level on children’s height-for-age z-scores (HAZs) and secondarily on stunting (HAZ < −2) and intermediary outcomes including household’s food insecurity, mother–child pairs’ diet and health, delivery in a health facility and low birth weight (LBW), women’s knowledge, and physical intimate partner violence (IPV).

**Methods and findings:**

We implemented a parallel-cluster–randomized controlled trial, in which 162 villages were randomized into either an intervention arm (UCTs + package of community activities, *n* = 82) or a control arm (package of community activities only, *n* = 80). Two different representative samples of children aged 6–29 months and their mothers were surveyed in each arm, one before the intervention in 2014 (control: *n* = 1,301, intervention: *n* = 1,357), the other 2 years afterwards in 2016 (control: *n* = 996, intervention: *n* = 1,035). Difference-in-differences (DD) estimates of impact were calculated, adjusting for clustering. Children’s average age was 17.4 (± 0.24 SE) months in the control arm and 17.6 (± 0.19 SE) months in the intervention arm at baseline. UCTs had a protective effect on HAZ (DD = +0.25 *z*-scores, 95% confidence interval [CI]: 0.01–0.50, *p* = 0.039), which deteriorated in the control arm while remaining stable in the intervention arm, but had no impact on stunting (DD = −6.2 percentage points [pp], relative odds ratio [ROR]: 0.74, 95% CI: 0.51–1.06, *p* = 0.097). UCTs positively impacted both mothers’ and children’s (18–23 months) consumption of animal source foods (ASFs) (respectively, DD = +4.5 pp, ROR: 2.24, 95% CI: 1.09–4.61, *p* = 0.029 and DD = +9.1 pp, ROR: 2.65, 95% CI: 1.01–6.98, *p* = 0.048) and household food insecurity (DD = −10.7 pp, ROR: 0.63, 95% CI: 0.43–0.91, *p* = 0.016). UCTs did not impact on reported child morbidity 2 week’s prior to report (DD = −3.5 pp, ROR: 0.80, 95% CI: 0.56–1.14, *p* = 0.214) but reduced the financial barrier to seeking healthcare for sick children (DD = −26.4 pp, ROR: 0.23, 95% CI: 0.08–0.66, *p* = 0.006). Women who received cash had higher odds of delivering in a health facility (DD = +10.6 pp, ROR: 1.53, 95% CI: 1.10–2.13, *p* = 0.012) and lower odds of giving birth to babies with birth weights (BWs) <2,500 g (DD = −11.8, ROR: 0.29, 95% CI: 0.10–0.82, *p* = 0.020). Positive effects were also found on women’s knowledge (DD = +14.8, ROR: 1.86, 95% CI: 1.32–2.62, *p* < 0.001) and physical IPV (DD = −7.9 pp, ROR: 0.60, 95% CI: 0.36–0.99, *p* = 0.048). Study limitations included the short evaluation period (24 months) and the low coverage of UCTs, which might have reduced the program’s impact.

**Conclusions:**

UCTs targeting the first “1,000 days” had a protective effect on child’s linear growth in rural areas of Togo. Their simultaneous positive effects on various immediate, underlying, and basic causes of malnutrition certainly contributed to this ultimate impact. The positive impacts observed on pregnancy- and birth-related outcomes call for further attention to the conception period in nutrition-sensitive programs.

**Trial registration:**

ISRCTN Registry ISRCTN83330970.

## Abstract 2

Justine Briaux and colleagues present work on child health and welfare following a trial of cash transfer to mothers.

## Author summary

**Why was this study done?:**

- Globally, 144 million children under 5 are still suffering from stunting, 40% of whom live in Africa. There is no simple solution to fight stunting; to address its multifactorial causes, a combination of nutrition-specific and nutrition-sensitive interventions is required.
- Cash transfer (CT) programs, which consist of direct cash payments to eligible beneficiaries, are among the promising nutrition-sensitive interventions, having demonstrated (mainly in Latin America and, more recently, in Eastern Africa) positive impacts on poverty, food security, attendance at health services, and, to a lesser extent, children’s health and nutritional status.
- Despite their great potential, CTs’ impact on child linear growth is not conclusive, and their pathways of impact are not clearly understood. Studies thoroughly examining CTs’ mechanisms are needed, especially in West Africa, where evidence of CTs’ impact is scarce and where the successes seen in Latin America may not be replicable because of specific constraints that may affect the West African family environment.

**What did the researchers do and find?:**

- The researchers randomly allocated 162 villages from 5 northern districts in Togo to either an intervention arm or a control arm. Although both study arms benefited from the integrated community case management of childhood illnesses and acute malnutrition (ICCM-Nut) and behavior change communication (BCC) activities related to children’s nutrition, health, and protection, only women from the intervention arm received in addition monthly unconditional cash transfers (UCTs) (US$8.40/month) during their child’s “first 1,000 days” of life (from pregnancy to their second birthday).
- Surveys were conducted at 2 points in time among different samples of mothers and their children, 6–29 months old, both before the intervention began (2014, *n* = 2,658) and 2 years later (2016, *n* = 2,031).
- Despite substantial implementation issues, researchers found a protective effect of UCTs combined with ICCM-Nut and BCC on children’s linear growth which significantly deteriorated in the control arm, whereas it remained stable in the intervention arm. They also found positive impacts on various intermediary outcomes along the program impact pathways, including household food insecurity, consumption of animal source food, delivery in a health facility, low birth weight, intimate partner violence, and women’s hygiene and knowledge.

**What do these findings mean?:**

- UCTs targeting the “first 1,000 days”, combined with BCC and ICCM-Nut, have the potential to improve children’s growth in at-risk populations of Togo. Their positive impacts on various program impact pathways also confirmed that in order to be efficient in the fight against stunting, interventions should address several determinants at a time.
- The positive impacts observed on pregnancy and birth-related outcomes reassert the importance of conception and preconception periods for children’s growth and call for further attention to that period in designing future nutrition-sensitive programs.
- To lift the remaining barriers that prevent CT programs from realizing their full potential in West Africa, future research should further focus on programs implementation, coverage and uptake as well as on contextual effect modifiers (sociocultural, socioeconomic, political and environmental factors) that may hinder or enhance impact. In addition, theory-driven evaluations, which precisely estimate the contribution of a wide range of mediating factors to the final impact on linear growth (e.g., diet quality and quantity, health and medical follow-up, hygiene and sanitation, women’s empowerment) are also needed to identify the most effective levers of actions and help design future programs.

## Introduction

Although the number of stunted children under age 5 has fallen in many countries over the past decades, global figures remain alarming, and stunting is declining too slowly to meet the target set by the World Health Assembly for 2025 [1]. In West and Central Africa, the number of stunted children has actually increased by 7.8 million between 2000 and 2019, and little improvement is predicted in the coming years [2]. This puts those children at great risk of impaired physical and cognitive development, poor health, reduced scholarly success, and in the longer term, low economic productivity and poor reproductive outcomes for girls [3,4]. The international nutrition community contends that effective strategies to reduce growth retardation lie in the adoption of multisectoral approaches, which combine nutrition-specific and nutrition-sensitive interventions targeted at the “first 1,000 days” of life (between conception and the child’s second birthday) [5–7].

Promising nutrition-sensitive interventions include cash transfer (CT) programs. Originating in Latin America, these programs proved to be effective in reducing poverty and food insecurity and in increasing school enrollment and use of health services [8]. Despite their great potential, CT programs have demonstrated relatively modest impacts on childhood nutritional outcomes (linear growth, growth retardation, and micronutrient status) [9–11]. “Cash plus” programs, which combine CTs with additional components (e.g., community education and nutritional supplements), tend to have a greater impact than cash alone, especially on longer-term outcomes such as knowledge and behavioral changes, morbidity, and nutrition [12]. Those results have contributed to the popularity and expansion of CT programs worldwide, as reflected by the number of reviews on the topic [8,13–20]. The most recent reviews integrate new and emerging evidence from sub-Saharan Africa, where the number of CT programs has considerably increased since the 2000s [9,21]. Established in 2008, the multicountry “Transfer Project” research initiative provided evidence on the effectiveness of large-scale national CT programs in sub-Saharan Africa and partly rectified the knowledge gap for this part of the world [22]. However, there is still a dearth of evidence in West Africa, where most CT programs were small-scale pilots relying on external funding. The few published impact evaluations from Mali, Burkina Faso, and Ghana did not demonstrate any positive impact of CT programs on nutritional outcomes (stunting or wasting) among young children [23–25].

Optimizing the nutritional impact of future programs will require a better understanding of how CT programs work [26]. Very few evaluations have thoroughly and systematically studied CTs’ mechanisms of action and documented the pathways through which impact on nutritional status was achieved. In that sense, documenting the impact of CT programs on intermediary outcomes along the program’s theoretical impact pathways is as important as documenting their impact on primary nutritional outcomes. In 2014, at the scale of 5 districts, the government of Togo implemented a “cash plus” program combining unconditional CTs (UCTs) with community activities (sensitization meetings and home visits directed at child health, nutrition, and protection, as well as integrated community case management of childhood illnesses and acute malnutrition [ICCM-Nut]) targeted at mother–child pairs during the “first 1,000 days.” The aim of the program was to improve children’s nutrition, health, and protection. We implemented a cluster-randomized controlled trial to assess the impact of this program on children’s linear growth and on multiple intermediary outcomes along the program’s theoretical impact pathways.

We hypothesized that the program would improve children’s growth through a synergy effect between the community activities, which would raise mothers’ awareness and provide them with essential knowledge in health and nutrition, and the cash that would allow them to put into practice what they have learned through those community activities. This would result in improved health and nutrition of mother–child pairs (increased health-seeking behavior, decreased morbidity, higher dietary diversity, increased consumption of nutrient-rich foods), which in turn would help improve children’s growth. Providing women with better knowledge and financial means would also contribute to their empowerment, which has been recognized as an important factor in their own health and that of their children.

## Methods

### Setting and intervention

The pilot CT program was administered for 30 months in 2014 by the government of Togo, with financial and technical support from the World Bank and UNICEF. It consisted of monthly cash distributions to women during their children’s first 1,000 days of life (5,000 XOF, i.e., US$8.30/month) combined with behavior change communication (BCC) activities (including home visits and community sensitization meetings) and with the integrated community case management of childhood illnesses and acute malnutrition (ICCM-Nut). The program was implemented in the 5 districts (Dankpen, Doufelgou, Keran, Oti, and Kpendjal) of the Kara and Savanes regions, where the highest rates of acute and chronic malnutrition in children under 5 exist. In 2014, 33.7% and 32.1% of children under 5 years of age were stunted in the Kara and Savanes regions, respectively, versus 27.5% at the national level. The same regions also saw rates of wasting in children of 7.2% and 11.2% versus 6% at the national level. Within the 5 aforementioned districts, the program was implemented in the villages where the “ICCM-Nut program” was running. Carried out since 2011 by the Ministry of Health and UNICEF, this program targeted rural landlocked villages that had poor access to health facilities. In those villages, community health workers (CHWs) were trained and equipped to screen and treat childhood malaria, diarrhea, pneumonia, and acute malnutrition and to lead sensitization meetings on essential family practices (e.g., exclusive breastfeeding, hand washing with soap, and health-seeking behavior). On top of those preexisting activities, BCC sessions focusing on child protection issues (e.g., birth registration, schooling, or fostering) were organized by specifically trained community child protection workers (CCPWs) together with the CTs (S1 Table). CHWs and CCPWs were chosen by the community on the basis of 4 prerequisites: (i) live in the village, (ii) be proficient in French (language used for the trainings), (iii) have the highest possible level of education (have attended at least primary school, know how to read and write), and (iv) be committed to children. Given the minimum educational requirements, most of them were men. The combination of CT and BCC activities (CHWs’ and CCPWs’ sensitization meetings and home visits) was expected to provide women with the essential knowledge and financial resources for the adoption of good childcare practices. CTs were unconditional, but recipient women were strongly encouraged to adopt specific behaviors conducive to their children’s protection and well-being, namely to fulfill at least 4 antenatal visits, register children’s births, enroll children in primary school, keep children younger than 15 at home (no fostering), and attend CCPW and CHW sensitization meetings. Women attending sensitization meetings with assiduity received a bonus of 20,000 XOF (approximately US$33) when exiting the program. To qualify for this reward, women were required to attend at least *m* + 1 sensitization meetings, where *m* is the mean number of times women of a given village took part in sensitization meetings.

### Participants

Women who were at least 3 months pregnant and mothers of children aged 0–23 months were eligible to receive the CT. No minimum age limit was set up. If eligible, adolescent women could also benefit from the CT. Women with several eligible children received the intervention for the youngest one only. However, mothers of twins and mothers with both an eligible biological child and an eligible fostered child received the CTs for both children. Mothers with a child aged 24–59 months suffering from severe acute malnutrition were also eligible to receive the CTs. Beneficiary women could enter the program anytime between their second trimester of pregnancy and their child’s second birthday throughout the program duration (knowing that the beneficiaries’ list would be updated every 2 months). Regardless of when they entered, they received the CTs for a minimum of 12 months. Those who entered early in pregnancy took full advantage of the program, benefiting from the CTs for the maximum duration of 30 months.

### Randomization

A nonblinded parallel-cluster–randomized controlled trial was carried out to assess the impact of the cash component of the program. A total of 162 villages were randomized into either a control arm benefiting from the ICCM-Nut program (ICCM-Nut and CHWs’ sensitization meetings around essential family practices) and from CCPWs’ package of activities (sensitization meetings and home visits around child protection issues) (*n* = 80 villages) or into an intervention arm benefiting from the ICCM-Nut program, CCPWs’ package of activities, and CTs (*n* = 82 villages). Initially, 81 villages were allocated to each arm, but 1 control village was accidentally allocated to the intervention arm while disseminating the randomization results to the communities; it was decided to maintain this village in the intervention arm for ethical reasons. The randomization was stratified by district and carried out by the government of Togo and the World Bank using a random real number, generated with the RAND function in Excel.

### Sampling

**Fig 1:**
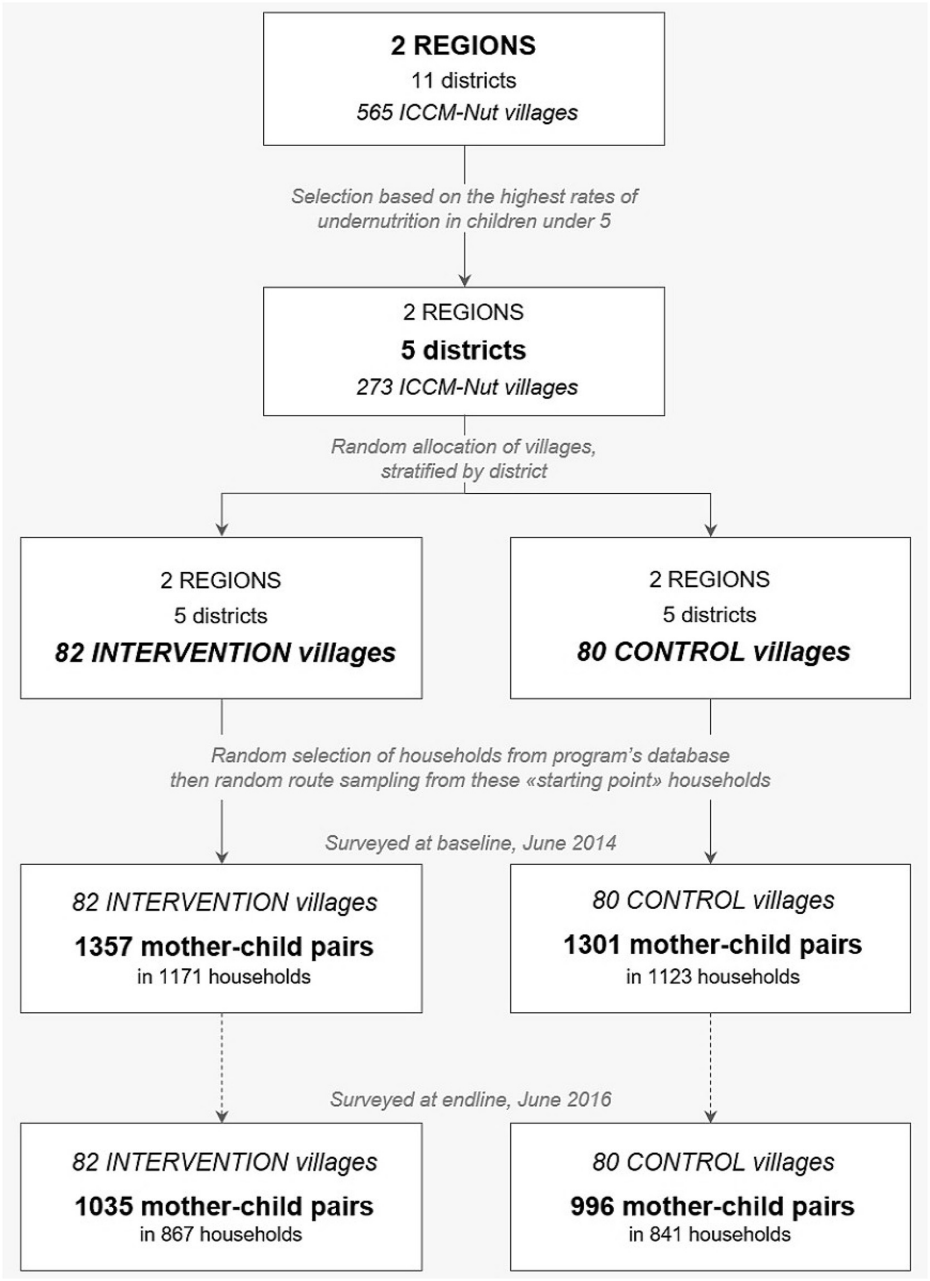
Flow chart of the study. CT cluster-randomized controlled trial, Northern Togo, 2014–2016. CT, cash transfer; ICCM-Nut, integrated community case management of childhood illnesses and acute malnutrition.

Two repeated cross-sectional surveys were conducted, one before the first distribution of cash (baseline survey, May 16 to July 4, 2014) and the other after 24 months of program operation (endline survey, May 26 to June 16, 2016) (Fig 1). Representative samples of mothers and their 6- to 29-month–old children in both arms of the study were surveyed in each village. This age range was the best compromise for reasonable theoretical exposure of children to the program (at least 12 months) and stage of exposure (during pregnancy or not later than 6 months of age during infancy) after 2 years of program operation (S1 Fig). In each village, households were randomly selected from the program census database. The latter was compiled prior to the beginning of the study through an exhaustive household census whose aim was to list the households eligible for CTs in each ICCM-Nut village, i.e., those with at least one pregnant woman or a child under 2 years of age. To avoid the overrepresentation of children aged 6–23 months in our sample, we used these households as “starting points” from which we applied a random-route sampling method. At each “starting point,” a household member rolled a die. When the die indicated 5 or 6, we included the household when eligible for the evaluation study (i.e., when at least one child aged 6–29 months was living in the household) or we included the nearest eligible household otherwise. If the die indicated 1, 2, 3, or 4, we systematically selected the eligible household living next to the “starting point” household. If several households were living next to the “starting point” household (at equal distances, in a same compound), one was randomly selected directly in the field. Within selected households, we surveyed all eligible mother–child pairs. If a mother had more than 1 eligible child, the enumerator randomly selected one. This sampling procedure was repeated identically between baseline and endline surveys.

The sample size was calculated to detect a change of 0.20 *z*-score in height-for-age (HAZ) among 6- to 29-month–old children while considering the following parameters: 5% significance level, 90% of power, 10% of missing/invalid data, 162 villages to be randomized in 2 arms, and an intraclass correlation coefficient of 0.02. To detect a 0.20 HAZ difference between the intervention and control arms at endline (assuming a variance of 1.3 determined from previous studies), a sample size of 1,020 children per arm and per survey was required. This sample size also allowed detecting a difference in the prevalence of stunting of 9% between the 2 arms. This calculation was conservative because it did not account for the district stratification used for randomization. An average of 13 mother–child pairs were surveyed in each village (*n* = 128), ranging from 5 pairs in small villages (*n* = 16) to 17 pairs in large villages (*n* = 18).

### Data collection

**Fig 2:**
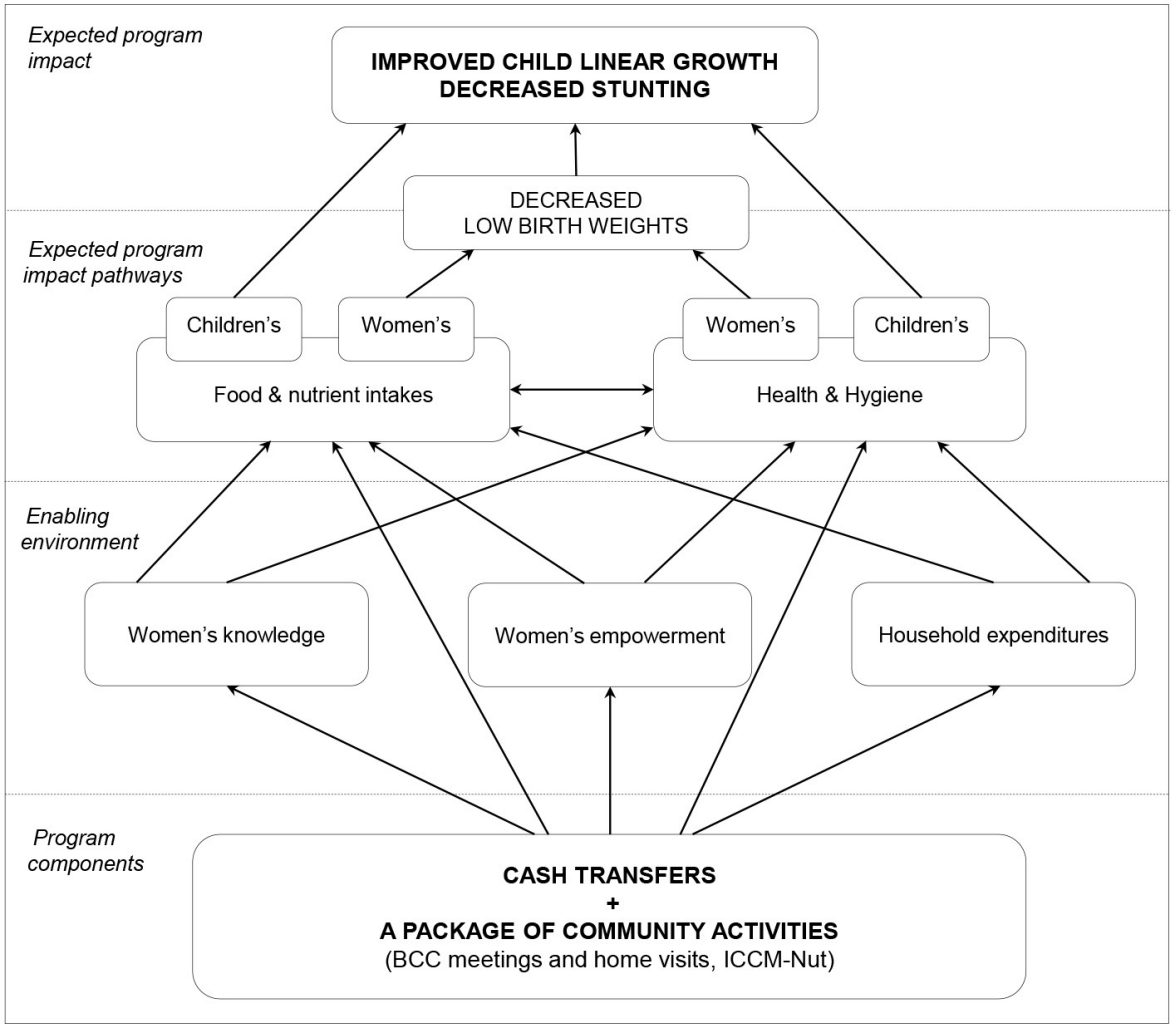
Simplified theoretical impact pathways of the program. CT cluster-randomized controlled trial, Northern Togo, 2014–2016. BCC, behavior change communication; CT, cash transfer; ICCM-Nut, integrated community case management of childhood illnesses and acute malnutrition.

Data were collected using the same standardized questionnaire at baseline and endline. The questionnaire was administered face-to-face to women and heads of households during home visits by experienced enumerators speaking the local languages of the study area. These enumerators were identified through a highly selective process and extensively trained over 2 weeks. Their training mainly consisted of the exhaustive review, translation, and pretest of the questionnaire; each question was standardized across the different vernacular languages spoken in the study area. Enumerators also learned how to perform anthropometric measurements and how to collect data through tablets. Built upon the program’s theory of change, the questionnaire covered a wide range of intermediary outcomes meant to document the program’s theoretical impact pathways, which are presented in their simplified version in Fig 2 (for a more comprehensive version, please see [27]). At endline, a complementary module was administered to women in order to document how the program rolled out and how it was used by beneficiaries and to collect information on potential unexpected effects.

### Primary and secondary outcomes

The impact was primarily evaluated on HAZ and secondarily on stunting (HAZ < −2 SD) among 6- to 29-month–old children. Children’s height measurement was standardized according to WHO recommendations [28] and carried out by specifically trained enumerators and assistants. The recumbent length of under-2 children and the standing height of older children were measured to the nearest millimeter using portable devices equipped with height gauges. Children’s ages were reported from official documents when available or from the mother’s memory, using a calendar of local events if necessary.

### Intermediary outcomes

We identified 2 major pathways whereby the program may impact children’s growth and several enabling factors that may facilitate the impact achievement (Fig 2).

#### The “food and nutrient” pathway

Indicators along this pathway included the Infant and Young Child Feeding (IYCF) practices indicators, namely the Minimum Dietary Diversity (MDD), the Minimum Meal Frequency (MMF), and the Minimum Acceptable Diet (MAD), which were computed among 6- to 23-month–old children following WHO guidance [29]. We also looked at dietary diversity scores (DDSs) among the whole sample of children, derived from a qualitative multiple-pass 24-h recall and using a 7-food–group classification (grains, roots, and tubers; legumes and nuts; dairy products; flesh foods; eggs; vitamin-A–rich fruits and vegetables; other fruits and vegetables). Likewise, a qualitative multiple-pass 24-h recall performed with mothers was used to compute a DDS for women (WDDS10) using a 10-food–group classification (grains, roots, and tubers; pulses; nuts and seeds; flesh foods; dairy; eggs; dark green leafy vegetables; other vitamin-A–rich fruits and vegetables; other vegetables; other fruits), as recommended by the FAO [30]. The WDDS10 of women of reproductive age (aged 15 to 49) was further dichotomized to compute the Minimum Dietary Diversity for Women (MDD-W) [30,31]. Optimal breastfeeding initiation was computed for children <24 months old (i.e., children who entered the program at birth or before birth). Breastfeeding initiation was considered optimal when 3 conditions were met: the child was given breastmilk within the hour after birth, received colostrum, and was not given any other liquids before initiating breastfeeding. We estimated food insecurity experienced by households over the previous 30 days using the standard Household Food Insecurity Access Scale (HFIAS) [32].

#### The “health and hygiene” pathway

At the child level, we collected data on overall health since birth (as perceived by the mother), morbid episodes over the previous 15 days, and medical monitoring since birth. At the mother level, we collected information on antenatal follow-up, delivery, and postnatal care. In children aged less than 20 months, who had a chance to be exposed to the program in utero, we collected birth weights (BWs) from health documents when available and calculated the proportion of children with low birth weight (LBW) (BW < 2,500 g). The hygiene of individuals and their houses was assessed using spot-check observations [33,34]. In this approach, a list of predetermined conditions (e.g., cleanliness of hands) is observed during a home visit. We considered good hygiene in children to mean their hands, face, and hair were clean (clothes were not taken into account because many children were naked). We deemed mothers’ hygiene good if their hands, face, and clothes were clean (hair was not observed because many mothers were not willing to remove their headscarves). We considered the hygiene of the house to be good if no garbage and no animal feces were observed in the yard. Overall hygiene was scored “good” if the hygiene of the children, their mother, and the yard were all individually rated “good.”

#### Enabling environment

See S2 Table. Mothers’ knowledge on breastfeeding, nutrition, child’s health, pregnancy, delivery, hygiene, and birth registration was assessed. Women were attributed points depending on the number of correct answers they provided in each domain; points were summed to calculate a global knowledge score, which was further categorized into terciles to identify women with poor, medium, or good global knowledge. Two dimensions of women’s empowerment were assessed: the decision-making power of women within their households and intimate partner violence (IPV). Decision-making is modeled after questions from the demographic and health surveys (DHSs) [35] and IPV after WHO’s Violence Against Women instrument (VAWI) [36]. One point was given when the woman made decisions on her own for each of the 12 different topics relating to her autonomy, social life, and care for her children. Points were summed to calculate a continuous decision-making score, which was further divided into terciles to identify women with low, moderate, or high decision-making power. Regarding IPV, we estimated the proportion of women who endured controlling behavior, emotional violence, or physical violence from their partners over the past 12 months. Household expenditure was captured through standardized questions similar to that of Household Budget Survey (HBS). Nonfood expenditure was estimated over the last month. Exceptional expenditures (funerals, weddings, religious feast) were removed from the calculation. Food expenditure, including self-consumption, was assessed on the previous day, month, or year according to the type of food considered and then reduced to the last month. Household self-consumption was measured through household utensils and turned into monetary value on the basis of domestic food prices, collected on the markets of the studied area. The average price of each food item was calculated from the prices charged by 3 different vendors. Then, both food and nonfood expenditures were divided by the number of individuals in the household to obtain monthly per capita expenditure.

#### Sociodemographic characteristics

We collected information on household composition and size, on the quality of the house (main source of drinking water, sanitation, main source of energy for cooking, number of rooms), and on the head of household’s sex, level of education, religion, and primary occupation. A youth ratio, defined as the number of household members <15 years of age over the number of persons ≥15 years, and a dependency ratio, defined as the number of people not contributing to household income over the number of people contributing to household income, were computed to account for household structure. Mothers’ ages, pregnancy statuses, education, and marital statuses were collected, as well as the child’s sex and age.

### Data management and statistical analysis

Data were collected using Android tablets (through ODK Collect at baseline and Survey CTO at endline), ensuring quality controls at data entry. Collected data were regularly sent to an online server, allowing additional quality checks and feedback for fieldwork improvement. Data quality was also guaranteed by the close field supervision of enumerators throughout the study. Data management was performed with R 3.3.2., and data analysis was performed using Stata 14.2. HAZs and stunting were computed using WHO’s multicenter growth reference standards macro for R [37]. All analyses were conducted using Stata’s svy commands to account for the sampling design (clusters, strata, and sampling weights).

#### Comparability of baseline characteristics and program coverage between arms

Comparability of the trial arms at baseline was tested on sociodemographic characteristics and anthropometric outcomes using linear regression models for continuous variables and logistic regression models for categorical variables. Using the same method, we also compared at endline the coverage of the BCC component between arms and provided a few descriptive statistics on the coverage of the CT component in the intervention arm.

#### Impact analyses

The program’s impact on primary and secondary outcomes (HAZ, stunting) was estimated using a difference-in-differences (DD) analysis. DD estimates were computed using linear regressions for HAZ and logistic regressions for stunting. The DD model can be specified in regression form as

$$Y_{i}=\beta 0+\beta 1\;phase+\beta 2\;arm+\beta 3\;phase\times arm+\beta 4\;X_{i}+\mu_{i},$$

where ***Y***_***i***_ is the outcome of interest, ***phase*** indicates the time of the survey (baseline/endline), ***arm*** indicates the intervention (CT + package of community activities/package of community activities only), ***phase*** × ***arm*** is the interaction term between the phase and the trial arm, ***X***_***i***_ is a matrix containing a set of control variables, ***μ***_***i***_ is the random unobserved error term, ***β*1** represents the time trend common to intervention and control arms ***β*2** accounts for the time-invariant differences between the intervention and control arms, ***β*4** is the vector of coefficients corresponding to the matrix of control variables, and ***β*3** is the DD estimator, which estimates the average program’s effect. For HAZ, the DD estimate is equal to the regression coefficient (***β*3**); for stunting, the DD estimate is based on predicted adjusted values obtained from the regression model by arm and survey. It is reported in percentage points (pp) along with the relative odds ratio (ROR) arising from the logistic regression. Beta and ROR are presented along with their 95% confidence interval (95% CI) and *p*-value.

The program’s impacts on intermediary outcomes within the “food and nutrient” and “health and hygiene” pathways and on enabling environment variables were estimated via the exact same procedures used for HAZ for continuous outcomes and stunting for categorical outcomes. For several outcomes, we restricted the analyses to a subsample according to the child’s age at endline and his/her theoretical exposure to the program (S1 Fig); e.g., the impact on pregnancy-related outcomes was restricted to children aged 6–19 months at endline because children outside this age range had not—or had not sufficiently—been exposed to the program in utero (S1 Fig).

To increase the precision of our estimates, we systematically included in our analyses the following covariates: the district in all impact analyses, the child’s age and sex when examining the impact of the program on HAZ and stunting, the child’s age when examining the impact of the program on health outcomes, the child’s sex and maternal height when examining the impact of the program on BW and LBW, and verification (yes/no) of the interviewee responses in a health document when examining the impact of the program on pregnancy-related outcomes.

Finally, as recommended when analyzing results of superiority trials, we primarily conducted impact analyses using an intention-to-treat (ITT) approach [38]. Because a high proportion of eligible women did not receive the cash due to implementation issues, we also ran per protocol (PP) analysis. However, we did not base any of our conclusions on PP results; we used such data only to underpin the discussion when relevant [39]. It should be noted that our PP analysis was only based on the CT component of the program. Consequently, we did not abandon any observations in the control arm, and in the intervention arm, we retained all women who received cash at least once (*n* = 400). All these analyses have been prespecified in the study protocol (S1 Protocol).

### Ethics

Ethical clearance was provided by the Ministry of Health of Togo and by the consultative ethic committee of the Institut de Recherche pour le Développement in France. Written informed consent was obtained from all mothers who took part in the study. The trial was registered in the ISRCTN registry under the reference 83330970.

## Results

### Participants’ characteristics at baseline

The number of households and mother–child pairs surveyed at baseline and endline is presented in Fig 1. There were no significant differences between the control and intervention arms at baseline in any of the sociodemographic and economic characteristics documented at the household, mother, and child levels (Table 1). Anthropometric indicators of children were also similar between the 2 arms at baseline. Households had a mean size of 8 persons and were mostly headed by men (>93%) with poor education. The main occupation of households was farming. Around half of households had access to improved sources of drinking water, and less than 5% had access to improved sanitation. Mothers from both arms were 29 years old on average, approximately 74% of them had no education, and almost all were engaged in a marital union, either monogamous (42%) or polygamous (53%). The children in our sample were 17 months old on average, and half of them were boys. The mean HAZ was −1.1 (0.06) in the control arm and −1.2 (0.06) in the intervention arm; the mean BW was slightly above 3,000 g in both arms.

**Table 1:** Baseline characteristics of households and mother–child pairs by treatment arm. CT cluster-randomized controlled trial, Northern Togo, 2014–2016.

	Control	Intervention	*p*-Value of the Difference
	Mean (SE) or %		
**Household level**	(*n* = 1,123)	(*n* = 1,171)	
Household composition			
Household size (number of members)	8.5 (0.25)	8.1 (0.13)	0.24
Number of children aged 0–5 y	2.7 (0.07)	2.5 (0.06)	0.11
Number of children aged 6–14 y	2.4 (0.11)	2.3 (0.08)	0.51
Number of adults (>15 y)	3.4 (0.10)	3.3 (0.04)	0.40
Youth ratio	1.6 (0.04)	1.6 (0.04)	0.41
Dependency ratio	1.8 (0.04)	1.7 (0.04)	0.14
Head of household sociodemographic characteristics			
Sex—male (%)	95.3	93.8	0.29
Education—none (%)	53.7	56.7	0.48
Religion (%)			0.66
Animist	60.4	62.2	
Muslim	11.0	12.7	
Christian	19.2	17.1	
None	9.5	8.0	
Primary occupation—farming (%)	95.2	93.1	0.73
Household’s economic characteristics			
Access to improved drinking water source (%)	50.3	55.4	0.44
Access to improved sanitation (%)	3.5	2.6	0.46
Main source of energy for cooking—biomass fuel (%)	95.4	94.0	0.33
Number of rooms in the house	3.5 (0.13)	3.4 (0.06)	0.49
Total monthly expenditures per capita (XOF)^1^	10,585 (0.03)	10,869 (0.04)	0.68
**Mother level**	(*n* = 1,301)	(*n* = 1,357)	
Birth mother^2^ (%)	99.0	99.4	0.24
Age (y)	28.7 (0.27)	29.3 (0.30)	0.15
Education—none (%)	74.4	75.3	0.84
Marital status (%)			0.35
Monogamous union	42.2	42.6	
Polygamous union	54.4	52.4	
Alone (widow, single, divorced/separated)	3.4	5.0	
Pregnant women (%)	8.7	9.9	0.38
Height (cm)	(*n* = 1,300)	(*n* = 1,357)	0.66
	159.5 (0.20)	159.6 (0.18)	
**Child level**	(*n* = 1,301)	(*n* = 1,357)	
Sex—male (%)	50.3	50.9	0.82
Age (mo)	17.4 (0.24)	17.6 (0.19)	0.58
Age groups (%)			0.41
6–11 months	26.7	24.3	
12–17 months	27.5	27.3	
18–23 months	24.2	27.4	
24–29 months	21.7	21.0	
Anthropometric characteristics	(*n* = 1,286)	(*n* = 1,342)	
HAZs	−1.1 (0.06)	−1.2 (0.06)	0.67
Stunting (HAZ < −2 SD) (%)	28.1	30.4	0.34
BW from a health document (g)	(*n* = 322)	(*n* = 258)	
	3,027 (30.5)	3,023 (47.7)	0.94
LBW (BW < 2,500 g) (%)	9.5	13.9	0.11

^1^Expenditures are expressed as geometric means (US$1 = approximately 600 XOF). They were log transformed to run the regressions.

^2^Biological mother (as opposed to tutor/guardian).

**Abbreviations**: BW, birth weight; CT, cash transfer; HAZ, height-for-age z-score; LBW, low BW; SE, standard error.

### Program implementation and coverage

The program underwent major implementation issues, specifically regarding its CT component. At endline (June 2016), 58% of eligible women from our sample said they never received any cash (*n* = 635) (Table 2). The main reason for this lies in a major delay in updating the recipient list. Although the plan of implementers was to include eligible women before the program began (September 2014) and then refresh the list every 2 months, in reality, the second round of inclusion did not happen until May 2015. Furthermore, at the time of our endline survey, women identified in 2015 (1 year prior) were still waiting for their beneficiary cards and had not yet received any CTs. Several factors combined to explain this substantial problem with program fidelity. One of the reasons lies in the complex institutional set-up of the program, which involved 4 different ministries—the Ministry of Social Action, the Ministry of Health, the Ministry of Grassroots Development, and the Ministry of the Post—that had difficulty communicating and working together. Interviews conducted with program implementers as part of the process evaluation also indicated a lack of activity planning and an unclear definition of roles between actors, as illustrated by these words from a program stakeholder: “The ball is thrown into my court and I throw it back and that’s where we are […] and the project comes to a standstill.” All these problems resulted in sometimes huge delays in program implementation, as seen in the integration of new beneficiaries. In that specific case, program implementers also condemned a high, suspicious increase in the number of beneficiaries in between the 2 censuses, citing the allure of cash as a catalyst. Because many children either did not have birth certificates attesting to their age or had a certificate with questionable dates procured long after birth, the validation of the beneficiary list (both by the community and by independent consultants) took much more time than expected. In the end, out of the women who did receive the CTs (*n* = 400), 235 recalled how many times and claimed that they received it 13 times on average (Table 2). The median amount received by beneficiaries over 24 months was 60,000 XOF (min: 10,000–max: 135,000), in a country where in 2016 the average monthly income was around 26,000 XOF, 1 kg of maize cost 100 XOF, and one chicken around 2,000 XOF.

**Table 2:** Program coverage and uptake based on retrospective data collected at endline. CT cluster-randomized controlled trial, Northern Togo, 2014–2016.

	Control	Intervention	Difference between Arms	
	Mean (± SE) or %	Mean (± SE) or %	OR [95% CI]; Ref.: control arm	*p*
**CTs**				
Women received the CT at least once for the child surveyed (%)		(*n* = 400)		
		42.0		
Mean number of times women had received the CT (over 24 months)		(*n* = 235)		
		13.1 (± 0.45)		
Women regularly sharing their CT (%)		(*n* = 400)		
		18.5		
**CCPWs’ BCC activities**				
CCPW organized sensitization meetings	(*n* = 996)	(*n* = 1,035)		
Every month	83.0	92.2	2.41 [1.51, 3.86]	<0.001
Not every month	6.7	3.3		
Never	10.3	4.5		
Women attended CCPW’s sensitization meetings	(*n* = 893)	(*n* = 992)		
Always	72.4	82.6	1.81 [1.32, 2.49]	<0.001
Often	16.4	10.9		
Sometimes/rarely	8.5	5.0		
Never	2.7	1.5		
Women’s motivations to attend always or often CCPW’s sensitization meetings	(*n* = 787)	(*n* = 932)		
To continue receiving the CT—yes (%)	1.98	45.3	41.10 [22.54, 74.95]	<0.001
To enter the program/start receiving the CT—yes (%)	26.3	22.1	0.79 [0.56, 1.14]	0.207
To acquire new knowledge—yes (%)	82.7	70.4	0.50 [0.36, 0.69]	<0.001
To receive the bonus—yes (%)	5.2	30.1	7.87 [4.49, 13.77]	<0.001
Women received at least 1 visit from the CCPW	(*n* = 996)	(*n* = 1,035)		
	53.1	72.1	2.29 [1.70, 3.07]	<0.001
Mean number of visits received since the program started	(*n* = 476)	(*n* = 671)		
	6.8 (± 0.55)	7.9 (± 0.52)	1.10 [−0.40, 2.59]	0.149
Time since the last visit	(*n* = 542)	(*n* = 738)		
<1 month	74.4	78.8	1.28 [0.91, 1.80]	0.149
1–3 months	22.5	18.7		
> 3 months	3.1	2.4		
**CHWs’ BCC activities**				
CHW organized sensitization meetings	(*n* = 996)	(*n* = 1,035)		
Every month	68.8	76.4	1.47 [1.01, 2.13]	0.044
Not every month	8.1	6.6		
Never	23.0	16.9		
Women attended CHW’s sensitization meetings	(*n* = 753)	(*n* = 844)		
Always	70.6	77.4	1.43 [1.07, 1.91]	0.016
Often	16.6	13.2		
Sometimes/rarely	10.4	7.6		
Never	2.5	1.8		
Women’s motivations to attend always or often CHW’s sensitization meetings	(*n* = 651)	(*n* = 764)		
To continue receiving the CT—yes (%)	2.0	41.6	34.68 [18.12, 66.40]	<0.001
To enter the program/start receiving the CT—yes (%)	24.9	20.3	0.77 [0.53, 1.12]	0.174
To acquire new knowledge—yes (%)	81.8	73.0	0.60 [0.41, 0.88]	0.009
To receive the bonus—yes (%)	5.4	31.7	8.14 [4.31, 15.36]	<0.001

**Abbreviations**: BCC, behavior change communication; CCPW, community child protection worker; CHW, community health worker; CI, confidence interval; CT, cash transfer; OR, odds ratio; SE, standard error.

In comparison to CTs, CCPWs’ BCC activities and ICCM-Nut activities were implemented without major issues. The ICCM-Nut activities were already well orchestrated, and CHWs were able to help less experienced CCPW’s on a daily basis. Moreover, those program components were less multisectoral than the CT component and only relied upon 2 ministries, the Ministry of Health for the ICCM-Nut component and the Ministry of Social Action for the CCPWs’ activities. Although the implementation of those components was smoother and did not lead to substantial issues with program fidelity, they were nonetheless better implemented in the intervention arm than the control arm. Both CCPWs’ and CHWs’ BCC activities were more frequent in the intervention villages than in control villages (Table 2). For instance, women from the intervention arm were more likely to have received at least 1 CCPW visit than those from the control arm (OR: 2.43, 95% CI: 1.49–3.96, *p* < 0.001). CCPWs from intervention villages were also more likely to organize sensitization meetings than those from control villages (OR: 2.41, 95% CI: 1.51–3.86, *p* < 0.001). The same was true for CHWs (OR: 1.47, 95% CI: 1.01–2.13, *p* = 0.044). In turn, women from the intervention villages had higher odds of always attending sensitization meetings organized by CCPWs (OR: 1.81, 95% CI: 1.32–2.49, *p* < 0.001) and CHWs (OR: 1.43, 95% CI: 1.07–1.91, *p* = 0.016) than women from the control villages.

### Impact on child’s linear growth and stunting

**Fig 3:**
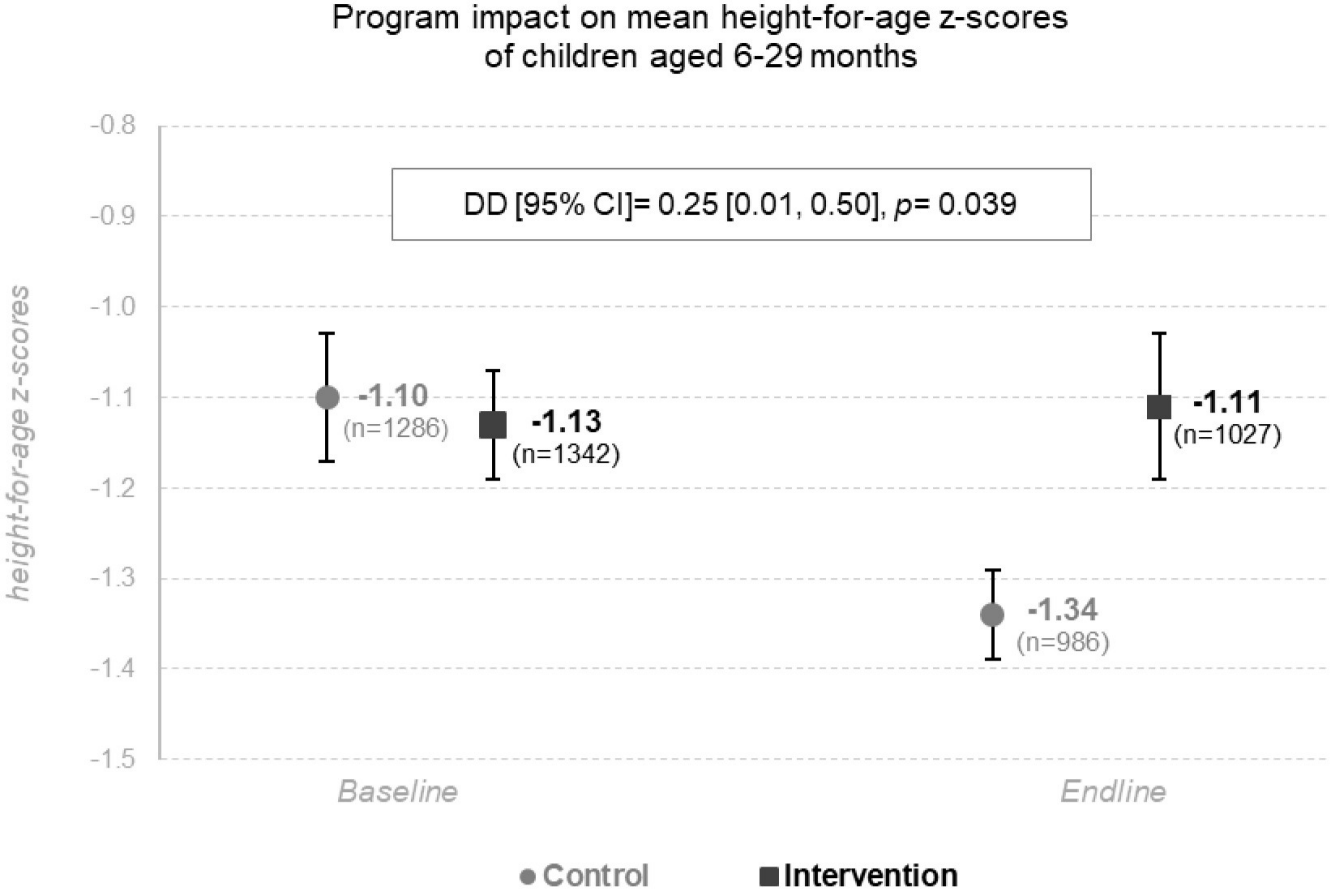
Mean HAZs ± SE of children aged 6–29 months at baseline (2014) and endline (2016) in the control arm and in the intervention arm, ITT analysis adjusted for child’s age, child’s sex, and districts. CT cluster-randomized controlled trial, Northern Togo, 2014–2016. CT, cash transfer; DD, difference-in-differences; HAZ, height-for-age z-score; ITT, intention to treat; SE, standard error.

The intervention had a significant impact on the linear growth of children 6-to 29-months old, resulting in a difference of 0.25 HAZ for the benefit of children from the intervention arm (DD = +0.25, 95% CI: 0.01–0.50, *p* = 0.039) (Fig 3). However, this DD was mainly due to a deterioration of HAZ in the control arm between baseline and endline surveys (−1.10 HAZ at baseline versus −1.34 HAZ at endline, β = −0.24 [−0.41, −0.07], *p* < 0.01), whereas HAZ remained stable in the control arm (−1.13 HAZ at baseline versus −1.11 HAZ at endline; β = 0.03 [−0.14, 0.21], *p* = 0.728). The impact found on HAZ did not apply to child stunting (DD = −6.2 pp, ROR: 0.74, 95% CI: 0.51–1.06, *p* = 0.097; S2 Fig).

### Impact on intermediary outcomes

#### The food and nutrient pathway

See Table 3. Globally, we observed no significant impact of the program on child feeding practices. However, when disaggregating the analyses by age groups, we found that older children (18–29 months) experienced positive impacts of the program on DDS7 (DD = +0.29, 95% CI: 0.03–0.54, *p* = 0.031). Children aged 18–23 months, who were still benefiting from the program at endline, also had higher odds of eating at least 2 types of ASF (DD = +9.1 pp, ROR: 2.65, 95% CI: 1.01–6.98; *p* = 0.048). The program also had a positive impact on the proportion of women who had at least 3 meals on the day prior to the survey (DD = +6.6 pp, ROR: 1.61, 95% CI: 1.07–2.40, *p* = 0.022) and on the proportion of women who consumed at least 2 types of ASF (DD = +4.5 pp, ROR: 2.24, 95% CI: 1.09–4.61, *p* = 0.029). No significant impacts were found on WDDS-10 or MDD-W. At the household level, the program had a positive impact on food security. Households from the intervention arm had lower odds of experiencing severe food insecurity than those from the control arm (DD = −10.7 pp, ROR: 0.63, 95% CI: 0.43–0.91; *p* = 0.016).

**Table 3:** Program’s impact on intermediary outcomes along the food and nutrient pathway. CT cluster-randomized controlled trial, Northern Togo, 2014–2016.

	Control		Intervention		Program’s Impact		
	Baseline	Endline	Baseline	Endline	*β*3 [95% CI]; ROR [95% CI]	DD	*p*
	% or mean (SE)		% or mean (SE)				
**Child feeding practices**							
Children aged 6–29 months	(*n* = 1,292)	(*n* = 992)	(*n* = 1,352)	(*n* = 1,024)			
Mean DDS7	2.8 (0.05)	2.8 (0.07)	2.8 (0.06)	2.9 (0.06)	0.09 [−0.17 to 0.34]	0.1	0.499
At least 2 types of animal source food (%)	5.8	5.9	6.0	10.2	1.75 [0.93 to 3.31]	4.1	0.082
Children aged 6–23 months	(*n* = 1,022)	(*n* = 807)	(*n* = 1,058)	(*n* = 816)			
MDD (%)	28.2	30.7	29.7	33.6	1.06 [0.71 to 1.59]	1.4	0.766
MMF (%)	74.9	78.3	74.1	77.1	0.94 [0.59 to 1.51]	1.0	0.809
MAD (%)	23.0	26.8	24.5	28.4	1.00 [0.67 to 1.49]	1.0	0.989
Optimal breastfeeding initiation (%)	(*n* = 931)	(*n* = 766)	(*n* = 960)	(*n* = 751)			
	35.6	39.5	35.7	44.8	1.24 [0.85 to 1.80]	5.2	0.265
**Maternal nutrition**							
All women	(*n* = 1,301)	(*n* = 996)	(*n* = 1,357)	(*n* = 1,035)			
At least 3 meals on the previous day (%)	83.1	81.9	81.1	86.5	1.61 [1.07 to 2.40]	6.6	0.022
Mean WDDS10	3.4 (0.04)	3.5 (0.06)	3.4 (0.05)	3.6 (0.06)	0.13 [−0.09 to 0.36]	0.1	0.233
At least 2 types of ASF (%)	4.5	4.5	4.2	8.7	2.24 [1.09 to 4.61]	4.5	0.029
Women of reproductive age (15–49 y)	(*n* = 1,291)	(*n* = 989)	(*n* = 1,352)	(*n* = 1,032)			
MDD-W (%)	13.8	18.8	16.6	22.2	0.99 [0.61 to 1.61]	0.6	0.982
**Household food insecurity**							
HFIAS (%)	(*n* = 1,123)	(*n* = 841)	(*n* = 1,171)	(*n* = 867)			
Severely food insecure	66.5	64.5	69.4	56.7	0.63 [0.43 to 0.91]	−10.7	0.016
Moderately food insecure	21.1	22.1	19.6	25.7		5.1	
Mildly food insecure	4.8	5.2	4.3	6.6		1.9	
Food secure	7.6	8.2	6.7	11.1		3.8	
HFIAS—continuous score	10.82	10.14	11.39	9.28	−1.43 [−2.59 to −0.28]	−1.4	0.015

**Abbreviations**: ASF, animal source food; CI, confidence interval; DD, difference-in-differences; DDS, dietary diversity score; DDS7, DDS with 7 food groups; HFIAS, Household Food Insecurity Access Scale; MAD, Minimum Acceptable Diet; MDD, Minimum Dietary Diversity; MDD-W, MDD for Women; MMF, Minimum Meal Frequency; ROR, relative odds ratio; SE, standard error; WDDS10, DDS for women with 10-food–group classification.

#### The health and hygiene pathway

See Table 4. Overall the program had no significant impact on children’s medical follow-up, except on the proportion of children who received vitamin A (DD = +6.7 pp, ROR: 1.65, 95% CI: 1.07–2.56, *p* = 0.024). The program had no impact on child morbidity (DD = −3.5 pp, ROR: 0.80, 95% CI: 0.56–1.14, *p* = 0.214). However, sick children from the intervention arm were less likely to forgo visits to the health center because of financial constraints than their control counterparts (DD = −26.4 pp, ROR: 0.23, 95% CI: 0.08–0.66, *p* = 0.006). The program had a positive impact on several antenatal care variables, including the proportion of women who received iron (DD = +4.5 pp, ROR: 2.08, 95% CI: 1.13–3.84, *p* = 0.019) and intermittent preventive treatment (IPT) for malaria (DD = +4.7 pp, ROR: 2.08, 95% CI: 1.04–4.16, *p* = 0.039). Cash-recipient women also had higher odds of delivering in a health facility as compared to nonrecipients (DD = +10.6 pp, ROR: 1.53, 95% CI: 1.10–2.13, *p* = 0.012). The program had a protective effect on LBW (DD = −11.8 pp, ROR: 0.29, 95% CI: 0.10–0.82, *p* = 0.020). Regarding postnatal care, beneficiary women were more likely to attend the postdelivery visit (DD = +12.9 pp, ROR: 3.28, 95% CI: 1.85–5.79, *p* < 0.001) and to receive postnatal iron supplementation (DD = +10.0 pp, ROR: 1.76, 95% CI: 1.20–2.59, *p* = 0.004) than nonbeneficiary women. The program had a positive impact on overall hygiene (DD = +9.0 pp, ROR: 1.44, 95% CI: 1.01–2.06, *p* = 0.046), which was mainly driven by the positive impact observed on women’s hygiene (DD = +9.0 pp, ROR: 1.54, 95% CI: 1.03–2.29, *p* = 0.035).

**Table 4:** Program’s impact on intermediary outcomes along the health and hygiene pathway. CT cluster-randomized controlled trial, Northern Togo, 2014–2016.

	Control		Intervention		Program’s Impact		
	Baseline	Endline	Baseline	Endline	*β*3 [95% CI]; ROR [95% CI]	DD	*p*
	% or mean (SE)		% or mean (SE)				
**Child health—*all children***							
Perceived health since birth—good (%)	(*n* = 1,301)	(*n* = 996)	(*n* = 1,357)	(*n* = 1,035)	1.39 [0.99 to 1.95]	4.6	0.058
	73.4	82.1	72.9	86.2			
Morbidity over previous 15 days—yes (%)	(*n* = 1,301)	(*n* = 996)	(*n* = 1,357)	(*n* = 1,035)	0.80 [0.56 to 1.14]	−3.5	0.214
	34.9	26.2	32.7	20.5			
Sick children (last 15 d) taken to a health center (%)	(*n* = 454)	(*n* = 270)	(*n* = 455)	(*n* = 234)	1.31 [0.81 to 2.13]	6.4	0.269
	57.9	64.5	54.4	67.4			
Sick children not taken to a health center because of lack of means (%)	(*n* = 199)	(*n* = 104)	(*n* = 193)	(*n* = 71)	0.23 [0.08; 0.66]	−26.4	0.006
	77.1	80.9	85.1	62.5			
**Child medical follow-up—*all children***							
Regular medical follow-ups since birth (%)	(*n* = 1,301)	(*n* = 996)	(*n* = 1,357)	(*n* = 1,035)	1.38 [0.83 to 2.31]	3.0	0.215
	79.6	91.3	78.3	93.0			
Age at last medical follow-up in months	(*n* = 551)	(*n* = 366)	(*n* = 573)	(*n* = 414)	0.66 [−0.05 to 1.38]	0.7	0.068
	10.7 (0.16)	10.5 (0.19)	10.5 (0.12)	11.0 (0.23)			
Full immunization—from a health document (%)^1^	(*n* = 839)	(*n* = 730)	(*n* = 832)	(*n* = 710)	1.17 [0.75 to 1.82]	3.8	0.480
	63.0	60.7	60.6	62.1			
Supplementation with vitamin A in the last 6 months (%)	(*n* = 1,275)	(*n* = 982)	(*n* = 1,312)	(*n* = 1,015)	1.65 [1.07 to 2.56]	6.7	0.024
	87.5	80.7	86.4	86.3			
Deworming in the last 6 months (%)	(*n* = 788)	(*n* = 573)	(*n* = 818)	(*n* = 639)	1.10 [0.60 to 2.03]	2.5	0.752
	71.5	58.7	72.4	62.1			
**Maternal antenatal care—*birth mothers of children aged 6–19 months***							
At least 1 antenatal consultation (%)	(*n* = 813)	(*n* = 644)	(*n* = 818)	(*n* = 673)	1.98 [0.92 to 4.25]	1.7	0.079
	97.6	97.1	96.9	98.1			
Mean number of antenatal consultations	(*n* = 724)	(*n* = 580)	(*n* = 701)	(*n* = 618)	0.12 [−0.17 to 0.40]	0.2	0.412
	4.0 (0.07)	3.8 (0.08)	3.8 (0.07)	3.8 (0.08)			
At least 4 antenatal consultations (%)	(*n* = 724)	(*n* = 580)	(*n* = 701)	(*n* = 618)	1.33 [0.87 to 2.01]	6.4	0.204
	64.6	55.7	60.7	58.2			
Stage of pregnancy at first antenatal visit in months	(*n* = 742)	(*n* = 586)	(*n* = 718)	(*n* = 634)	−0.25 [−0.61 to 0.11]	−0.2	0.173
	4.5 (0.07)	4.5 (0.09)	4.5 (0.08)	4.2 (0.11)			
Received iron (%)	(*n* = 799)	(*n* = 617)	(*n* = 782)	(*n* = 641)	2.08 [1.13 to 3.84]	4.5	0.019
	94.4	92.0	92.5	94.6			
Received intermittent preventive treatment for malaria (%)	(*n* = 790)	(*n* = 617)	(*n* = 774)	(*n* = 641)	2.08 [1.04 to 4.16]	4.7	0.039
	91.3	92.0	89.8	95.2			
Received tetanus vaccine (%)	(*n* = 791)	(*n* = 623)	(*n* = 783)	(*n* = 647)	1.83 [0.93 to 3.59]	3.8	0.078
	94.4	92.0	92.5	93.9			
Slept under an impregnated mosquito net during pregnancy (%)	(*n* = 813)	(*n* = 641)	(*n* = 815)	(*n* = 670)	2.67 [1.37 to 5.19]	7.0	0.004
	92.4	89.6	90.5	94.7			
**Delivery—*birth mothers of children aged 6–23 months***							
Delivery assisted by SBA (%)	(*n* = 1,018)	(*n* = 800)	(*n* = 1,057)	(*n* = 820)	1.44 [1.02 to 2.04]	9.1	0.041
	48.2	47.4	45.2	53.5			
Delivery in a health facility and assisted by an SBA (%)	(*n* = 1,018)	(*n* = 800)	(*n* = 1,057)	(*n* = 820)	1.53 [1.10 to 2.13]	10.6	0.012
	42.1	39.4	41.2	49.1			
Women who did not deliver in a health facility because of lack of means (%)	(*n* = 87)	(*n* = 72)	(*n* = 132)	(*n* = 49)	0.32 [0.10 to 0.99]	−19.1	0.048
	75.3	84.8	80.3	70.7			
**Newborns’ weight—*children aged 6–19 months***							
BW in grams recorded in a health document	(*n* = 210)	(*n* = 214)	(*n* = 176)	(*n* = 216)	137 [−42 to 316]	137.0	0.133
	3,023 (38.6)	2,984 (44.8)	2,975 (46.8)	3,072 (56.1)			
LBW (%)	(*n* = 210)	(*n* = 214)	(*n* = 176)	(*n* = 216)	0.29 [0.10 to 0.82]	−11.8	0.020
	9.2	11.4	16.3	6.7			
**Postnatal care**							
In birth mothers of children aged <25.5 mo^2^:	(*n* = 1,108)	(*n* = 846)	(*n* = 1,146)	(*n* = 872)	3.28 [1.85 to 5.79]	12.9	<0.001
At least 1 postnatal consultation—yes (%)	87.6	86.9	80.5	92.7			
In birth mothers of children aged <27 mo^3^:	(*n* = 1,098)	(*n* = 876)	(*n* = 1,123)	(*n* = 880)	1.76 [1.20 to 2.59]	10.0	0.004
Received iron—yes (%)	75.5	75.2	72.1	81.8			
**Child, maternal, and environmental hygiene—*all women and children***							
Child’s hands, face, and hair clean (%)	(*n* = 1,205)	(*n* = 953)	(*n* = 1,247)	(*n* = 968)	1.35 [0.88 to 2.06]	7.0	0.163
	49.5	62.1	47.9	67.5			
Mother’s hands, face, and clothes clean (%)	(*n* = 1,301)	(*n* = 996)	(*n* = 1,357)	(*n* = 1,035)	1.54 [1.03 to 2.29]	9.0	0.035
	54.2	64.6	54.8	74.2			
No waste or animal feces in the yard (%)	(*n* = 1,285)	(*n* = 985)	(*n* = 1,322)	(*n* = 1,017)	1.23 [0.86 to 1.76]	4.7	0.262
	48.5	57.5	51.5	65.2			
Overall hygiene (%)	(*n* = 1,193)	(*n* = 943)	(*n* = 1,216)	(*n* = 951)	1.44 [1.01 to 2.06]	9.0	0.046
	25.4	33.9	27.0	44.5			

^1^According to the Togolese immunization schedule: for children <9 months, BCG + OPV3 + Pentavalent3 (DTC-Hep B-Hib); for children ≥ 9 months, BCG + OPV3 + Pentavalent3 + VAR antimeasles vaccine + yellow fever vaccine.

^2^Within 6 weeks after delivery.

^3^After delivery, iron supplementation should be provided for at least 3 months.

**Abbreviations**: BCG, bacillus Calmette-Guérin (antituberculosis vaccine); BW, birth weight; CI, confidence interval; CT, cash transfer; DD, difference-in-differences; DTC, diphtheria-tetanus-pertussis; Hep B, Hepatitis B; Hib, Haemophilus influenzae type b; LBW, low BW; OPV3, oral polio vaccine 3 doses; ROR, relative odds ratio; SBA, skilled birth assistant; SE, standard error; VAR, varicella (chicken pox) vaccine.

#### Enabling environment

See Table 5. We observed a positive impact of the program on the global knowledge of women (DD = +14.8, ROR: 1.86, 95% CI: 1.32–2.62; *p* < 0.001) but a rather negative impact on their decision-making power (DD = −0.53, 95% CI: −1.01 to −0.05, *p* = 0.030). This result is mainly driven by a negative impact on their decision-making pertaining to children (DD = −0.31, 95% CI: −0.54 to −0.08, *p* = 0.008). Regarding IPV, women receiving the CTs had lower odds of having experienced physical violence in the last 12 months than nonbeneficiaries (DD = −7.9 pp, ROR: 0.60, 95% CI: 0.36–0.99, *p* = 0.048). We did not find any impact on controlling behavior or emotional violence. In line with the consumption of ASFs among women and children, the program had a positive impact on the amount that households spent at market to buy ASFs (DD = +619, 95% CI: 268–971, *p* = 0.001).

**Table 5:** Program’s impact on enabling factors. CT cluster-randomized controlled trial, Northern Togo, 2014–2016.

	Control		Intervention		Program’s Impact		
	Baseline	Endline	Baseline	Endline	*β*3 [95% CI]; ROR [95% CI]	DD	p
	% or mean (SE)		% or mean (SE)				
**Women’s overall knowledge**							
Overall knowledge (%)	(*n* = 1,301)	(*n* = 996)	(*n* = 1,357)	(*n* = 1,035)	1.86 [1.32 to 2.62]		<0.001
good	30.0	46.1	27.5	58.4		14.8	
average	36.7	33.9	36.4	28.3		−5.3	
poor	33.3	20.0	36.1	13.2		−9.6	
Continuous score (scored out of 44 points)	19.5 (0.24)	21.8 (0.32)	19.1 (0.30)	23.4 (0.26)	1.95 [0.92 to 2.98]	2.0	<0.001
**Women’s empowerment**							
Women’s decision-making (%)	(*n* = 828)	(*n* = 736)	(*n* = 851)	(*n* = 786)	0.76 [0.57 to 1.01]		0.059
high	31.3	36.9	35.7	35.1		−6.2	
moderate	28.7	28.9	28.9	28.9		−0.2	
low	40.0	34.2	35.4	36.0		6.4	
Continuous scores							
Global score—all 12 items	2.89 (0.09)	3.35 (0.16)	3.22 (0.16)	3.16 (0.10)	−0.53 [−1.01 to −0.05]	−0.5	0.030
Children’s nutrition, health, and education—5 items	0.98 (0.05)	1.24 (0.08)	1.20 (0.07)	1.14 (0.05)	−0.31 [−0.54 to −0.08]	−0.3	0.008
Women’s health, family planning, and pregnancy—3 items	0.52 (0.03)	0.62 (0.04)	0.63 (0.05)	0.61 (0.04)	−0.12 [−0.30 to 0.06]	−0.1	0.183
Women financial autonomy and freedom of movement– 4 items	1.39 (0.04)	1.44 (0.07)	1.48 (0.05)	1.38 (0.04)	−0.15 [−0.34 to −0.04]	−0.2	0.129
IPV—over the last 12 months							
Controlling behavior—yes (%)	(*n* = 899)	(*n* = 775)	(*n* = 941)	(*n* = 815)	0.93 [0.64 to 1.35]	−2.3	0.686
	72.5	63.9	69.1	58.2			
Emotional IPV—yes (%)	(*n* = 903)	(*n* = 767)	(*n* = 953)	(*n* = 818)	0.83 [0.56 to 1.25]	−3.6	0.374
	55.1	37.0	51.4	29.7			
Physical IPV—yes (%)	(*n* = 912)	(*n* = 778)	(*n* = 958)	(*n* = 831)	0.60 [0.36 to 0.99]	−7.9	0.048
	26.9	21.8	28.1	15.1			
**Household monthly per capita expenditures**^1^ **in XOF**							
Total expenditures (food + nonfood)	(*n* = 1,123)	(*n* = 841)	(*n* = 1,171)	(*n* = 867)	1,012 [−225 to 2,245]	1,012	0.108
	10,580 (0.03)	10,584 (0.03)	10,864 (0.03)	11,880 (0.03)			
Nonfood expenditures	(*n* = 1,123)	(*n* = 841)	(*n* = 1,171)	(*n* = 867)	487 [64 to 889]	487	0.022
	2,311 (0.04)	1,914 (0.04)	2,216 (0.05)	2,306 (0.05)			
Food expenditures	(*n* = 1,123)	(*n* = 841)	(*n* = 1,171)	(*n* = 867)			
Total expenditures (market + self-consumption)	7,333 (0.03)	7,653 (0.04)	7,676 (0.04)	8,579 (0.03)	583 [−416 to 1,583]	583	0.248
Market purchase only	3,421 (0.04)	3,187 (0.05)	3,462 (0.05)	3,746 (0.04)	518 [0 to 1,036]	518	0.050
Self-consumption only	1,179 (0.14)	1,396 (0.13)	1,245 (0.15)	1,303 (0.14)	−159 [−250 to 568]	−159	0.564
Focus on ASF expenditures	(*n* = 1,123)	(*n* = 841)	(*n* = 1,171)	(*n* = 867)			
ASF expenditures (market + self-consumption)	485 (0.16)	408 (0.15)	498 (0.14)	687 (0.17)	266 [−22 to 559]	266	0.071
Market purchase of ASF	742 (0.12)	549 (0.10)	765 (0.13)	1,191 (0.11)	619 [268 to 971]	619	0.001
Self-consumption of ASF	31 (0.20)	24 (0.19)	35 (0.16)	47 (0.21)	19 [−4 to 42]	19	0.100

^1^Expenditures are expressed as geometric means (US$1 = approximately 600 XOF). They were log transformed to run the regressions.

**Abbreviations**: ASF, animal source food; CI, confidence interval; CT, cash transfer; DD, difference-in-differences;; IPV, intimate partner violence; ROR, relative odds ratio; SE, standard error.

## Discussion

Our study showed UCTs associated with community activities (including ICCM of childhood illnesses and BCC activities related to children’s health, nutrition, and protection) and implemented over the “first 1,000 days” in Togo had a protective effect on linear growth among 6- to 29-month–old children when compared with community activities only. The program also had positive impacts on several intermediary outcomes along the “food and nutrient” pathways, those being household food security and consumption of ASFs by women and older children (18–23 months); along the “health and hygiene” pathway, positive impacts were seen on prenatal and postnatal iron supplementation, the proportion of women receiving IPT for malaria and sleeping under an impregnated mosquito net during pregnancy, the percentage of deliveries at health services, the proportion of LBW (among 6- to 19-month–old children), the proportion of women attending at least 1 postnatal consultation, and the hygiene of the mother. In the enabling environment, the program had a negative impact on women’s decision-making power but positive impacts on women’s knowledge, physical IPV experienced by women over the past 12 months, and household purchases of ASFs at markets. All of these impacts were found despite a suboptimal coverage of the CT component of the program.

The impacts of CT programs on nutritional outcomes have been reported in meta-analyses and reviews [9–11,19], but none of them found conclusive evidence regardless of the indicators considered. Most of the data came from conditional CT studies conducted in Latin America, even though evidence recently emerged from sub-Saharan Africa, including a small handful of West African countries, where no impact was found on nutritional outcomes. In Togo, the program had a positive impact on HAZ and LBW (in 6- to 19-month–old children). Although the positive impact of the program on LBW was clearly due to an improvement in the intervention arm, its impact on mean HAZ was mainly driven by a deterioration in the control arm, in which children surveyed at endline had lower HAZ than those surveyed at baseline. We assumed that this deterioration was mainly due to a bad conjuncture between 2014 and 2016, and that without the CTs’ distribution, the same thing would have happened in the intervention arm. A few elements in our data bolster that assumption. Looking at a series of shocks (disease, job loss, crop failure, etc.) households might have experienced over the past 12 months, we found that households had higher odds of having experienced losses of livestock at endline than at baseline in both the intervention (OR: 1.56, 95% CI: 1.26–1.93, *p* < 0.001) and control arms (OR: 1.40, 95% CI: 1.05–1.85, *p* < 0.05). In the intervention arm, however, households were less likely to report a high impact of those losses on their economic situation at endline than at baseline (OR: 0.51, 95% CI: 0.37–0.70, *p* < 0.001), pointing to a protective effect of the CTs. Moreover, given the low coverage of the program, the effect of the CTs in the intervention arm may also have been mitigated by children who never received it. PP analysis, even if exploratory, shows that CTs not only prevented a deterioration in children’s HAZ but actually improved it (DD = 0.62, 95% CI: 0.36–0.88, *p* < 0.001, S3 Fig). Those results are also corroborated by the PP results found on stunting (DD = −12.0 pp, ROR: 0.52, 95% CI: 0.35–0.77, *p* = 0.001; S2 Fig). The positive results found for both HAZ and LBW may be explained by several factors associated with the program. By targeting the 5 districts of the country with the highest rates of acute and chronic malnutrition in children under 5 and then in those districts selecting the villages with the least access to health services, the program had greater chances of success. Some studies have indeed shown that it is easier to provide meaningful changes in at-risk communities than in the overall population, in which the potential for improvements is lower [10–11]. On the other hand, adressing stunting through a single intervention in such at-risk communities, in which the issue is due to multiple and entangled factors (poverty, food insecurity, infectious diseases, limited access to basic services) may be more challenging than in other contexts, in which there are fewer contributory factors. Positive results seen on HAZ and LBW could also be explained by the fact that the program specifically targeted mother–child pairs during the “1,000 days” window of opportunity for addressing growth retardation and other forms of malnutrition, which probably maximized its impact [10, 26]. The transfer size of the program was also significant for this area. The literature suggests that transfers ranging from 15% to 25% of total monthly household expenditure are more likely to have impacts [10, 40]. In this study, we estimate that the transfers represented a minimum of 25%–30% of the total monthly expenditure of woman–infant pairs, even though it is very likely that part of the funds also benefited the entire household. The fact that CTs were combined with intensive BCC activities relating to health, hygiene, nutrition, and child protection certainly contributed to the success of the program by increasing participants’ awareness and knowledge of key childcare practices. During home visits, frontline workers provided individual psychosocial support and counseling on how to use the cash, which may have increased beneficiaries’ self-confidence and optimized the use of cash towards the adoption of such practices. Moreover, our results on program coverage suggest that the distribution of UCTs affected the service provision, as BCC activities seemed to be better implemented in the intervention arm than in the control arm. This certainly explains part of the impacts on HAZ and intermediary outcomes (consumption of ASF, maternal hygiene, maternal knowledge). This differential implementation may be due to higher motivation of frontline workers in the intervention villages. Because no blinding of the intervention was possible here, frontline workers may have been influenced by the knowledge of the allocation. Knowing that they were part of the intervention arm, they may have been more prone to be involved in their job because they would assume that families might be able to make better use of their recommendations thanks to the CT and also because they would feel watched by the program’s implementers and funders, who were awaiting results.

Regarding intermediary outcomes, we observed that the “cash plus” program increased households’ disposable income and that they used the cash to purchase food at markets, particularly ASFs. This resulted in a decrease in households’ experienced food insecurity, echoing the findings from 8 CT programs in sub-Saharan Africa (Ethiopia, Kenya, Lesotho, Malawi, Mozambique, South Africa, Uganda, and Zambia), as reviewed in De Groot and colleagues [6], and 1 food voucher program in Senegal [41]. This also led to higher consumptions of ASFs in older children and mothers, but not in dietary diversity indicators at the individual level or in other standard IYCF indicators. Evidence of CT programs’ impact on individual dietary indicators is limited, probably because intrahousehold allocation of foods may not be favorable to all members in several contexts. Furthermore, in our study we used qualitative indicators to reflect individuals’ diets, which may not be sensitive enough to detect significant changes [42,43].

Within the “health and hygiene” pathway, the program had no impact on children’s health or on children’s medical follow-ups, which were already quite good in both groups at baseline. In the literature, the effects of CTs on children’s health status are mixed, with some studies showing reduction in the prevalence of diarrhea or reduction in episodes of illness and other studies showing no impact at all [18]. Nevertheless, we showed that the program helped overcome the financial barrier for seeking healthcare for children in case of illness. Likewise, in Malawi, beneficiaries of the Mchinji Social CT program were more likely to receive care when sick compared with nonbeneficiaries [44]. The intervention also positively affected maternal antenatal and postnatal care, as well as the proportion of deliveries at health facilities and the proportion of LBW babies, which confirms the benefit of targeting women in early pregnancy, as shown in other studies from Latin America, Nepal, and India (reviewed in [45]).

Regarding the enabling variables, our findings showed, contrary to what could have been expected, that the program rather had a negative impact on women’s decision-making, at least when considering a score based on sole decisions by women. Taking a closer look at data, we showed that this result was mainly due to a decrease in beneficiaries’ decision-making power regarding decisions relative to children (such as health, nutrition, and schooling). Interviews with beneficiary women and their husbands, conducted as part of the process evaluation, indeed showed that fathers interfered more in decisions on children since the start of the program because they wanted to ensure "proper" use of the CTs. However, this does not mean that women had no say. Besides, the validity of decision-making measures have been criticized [46]; for example, the concept of “sole” versus “joint” decisions—potentially with or without disagreement—and how individuals perceive the notion of “ultimate decision maker” is difficult to capture quantitatively. Regarding IPV, women from the intervention arm had lower odds of being physically assaulted by their partner than women in the control arm. Very few studies looked at the impact of CTs on IPV, and to our knowledge, none of these were conducted in West Africa. These studies usually showed that CTs reduced physical and/or sexual IPV, mostly through an economic security and emotional well-being pathway [47,48]. Although the intervention had no overall impact on psychological/emotional violence, it had a positive impact on the proportion of women who were humiliated by their partner (DD = −6.4 pp, ROR: 0.61, 95% CI: 0.39–0.96, *p* = 0.031). Finally, the impact found on women’s knowledge could be explained not only by a better implementation of the BCC activities in the intervention arm, as already mentioned, but also by the strong motivations of beneficiaries themselves. They were incentivized both by the bonus and by the untrue belief that they needed to attend sensitization meetings to continue receiving the CTs.

Nevertheless, improving women’s awareness and lifting financial constraints may not always be sufficient. Demand-side interventions such as CTs should also invest in the supply side to be fully efficient. If the cash incentive allows women access to health services and food markets, then both quality healthcare and nutrient-rich foods should also be available to enhance their health and diet. To improve their impact, CT programs should also tackle issues that may optimize their implementation and coverage, such as communication and work sharing between stakeholders, staff organization and management, and planning and delivery of activities. Our study indeed showed that in remote and resource-poor settings, implementing such complex multisectoral programs according to protocol and ensuring their full coverage was challenging. This was especially true here given that the program was a pilot and that most of its operators were inexperienced. The program’s results could have been maximized through further experience, but although it was temporarily extended to other districts based on its positive results, the program did not continue in its current configuration. Nevertheless, it opened the road for CTs in Togo, as evidenced by the recent creation of a governmental CT program to support those who have lost their income because of the adoption of response measures against COVID-19. Moving beyond pilot and emergency programs to implement long-lasting and large-scale social safety nets will require more time and experience, but Togo now seems to be on the right track.

One major strength of our study lies in the use of a well-designed randomized controlled trial, in which we documented the program impact on a wide range of intermediary outcomes along the hypothesized program impact pathways, in addition to the impact on mean HAZ. Providing a global picture of the program impact rather than fragmented results helped in understanding the results found on linear growth. As foreseen in the theory of change of the program, our analyses confirmed that the intervention had an impact on multiple factors acting at different times over the “1,000 days” course to influence children’s growth. Examining the relative effect of each factor over the entire course of the intervention would have required performing a mediation analysis on the subsample of children aged 18–19 months, i.e., children who took full advantage of the intervention from early pregnancy to age 2. Unfortunately, our study was not powered for this. Besides, using a longitudinal design rather than repeated cross-sectional surveys would have allowed examining the change in children’s growth charts between groups to document the incidence of stunting rather than its prevalence. Because stunting results from a cumulative process, a longitudinal design would have provided more insight on children who were in the process of becoming stunted. Longitudinal designs may also offer opportunities for dose–response analyses. On the other hand, the usual delays in program roll-out and full implementation can have negative effects on an evaluation study cohort (e.g., children aging out of the “window of opportunity” for maximum nutritional impact of the intervention), making it unusable for assessing impact on age-sensitive outcomes such as stunting. Additional constraints of longitudinal designs include the logistical complexity and related additional cost of tracking individual children over time, with high risk of loss to follow-up. Finally, an information bias may also occur because individuals from a cohort are followed more closely than the whole population, with possible consequences on representativeness and external validity, whereas cross-sectional design better preserves real-life conditions.

This impact evaluation had some limitations. Although the program was running for 30 months, the impact study had to be conducted over a relatively short period of time—24 months—to avoid any seasonal effect between baseline and endline surveys. Theoretically, this time lapse was largely manageable in terms of the program’s correct functioning; nevertheless, there were still major implementation issues at the time of the endline survey, which probably weakened the impact of the program. This encouraged us to use PP analyses to discuss our results on children’s linear growth and stunting. Although this helped to confirm some ITT trends, we are aware that PP analysis is not without bias, especially because our PP analyses only concerned the CT component of the program and did not deal with participation in BCC activities. Because monitoring data were not made available to us, our analysis of program coverage is only based on retrospective declarative data collected from interviewees at endline. Such data are subject to recall bias. Beneficiary women may have been more likely than nonbeneficiaries to remember the number of times frontline workers organized sensitization meetings in their village or visited them. Moreover, we did not collect data on the functioning of health facilities, which could have helped explain certain results. Another limitation lies in the fact that the program itself may have influenced some interviewee’s responses, either because they wanted to stay in or because they wanted to make a good impression as beneficiaries. We attempted to mitigate such social desirability bias through the extensive training of enumerators. We are confident in the fact that our enumerators were trained enough to minimize this bias, however. Lastly, the external validity of our study is limited to rural at-risk areas of Western African countries.

To conclude, we demonstrated that CTs implemented during the “first 1,000 days,” in combination with community activities (including BCC sessions, home visits, and ICCM-Nut), had a protective effect on child’s growth among young children living in a vulnerable area of northern Togo. Our study contributes to the existing literature on CTs by providing new evidence from a West African country where data are missing and fills a gap by examining the effect of CTs on multiple intermediary outcomes along the theoretical impact pathways. Besides, the impacts found on various factors along those pathways confirm that to be efficient in the fight against stunting, interventions should address several determinants at a time and give particular attention to the conception and preconception periods, as evidenced by the positive effects observed on pregnancy- and birth-related outcomes. Our study also suggests that to impact growth, CT program should meet certain conditions including articulating clear nutrition objectives, providing sufficient amounts of cash, targeting the most at-risk populations over the first 1,000 days, and combining CTs with supply-side investments and educational components. It indeed supports the view that moving toward “cash plus” programs—i.e., programs that bundle CTs with additional components in an effort to improve the effect of the money—is key to making social protection truly transformative [49]. To be fully conclusive on this matter, studies comparing CT only with CT+ additional components such as BCC would be necessary. Researchers should also develop theory-driven evaluations that precisely estimate the contribution of a wide range of mediating factors to the final impact on linear growth (e.g., diet quality and quantity, health and medical follow-up, hygiene and sanitation, women’s empowerment) using, for instance, mediation analysis or structural equation modeling. This would help design future CT programs by identifying the most effective levers of actions to improve child’s growth. Finally, rigorous process evaluations addressing issues relating to CT programs’ implementation, coverage, and uptake are also needed to help lift the remaining barriers that prevent CTs from realizing their full potential.

## Supporting information

S1 Fig Stage and duration of children’s exposition to the program and justification of subsample analyses according to exposition. CT cluster-randomized controlled trial, Northern Togo, 2014–2016. CT, cash transfer.
(TIFF)

S2 Fig Proportion of stunted children aged 6–29 months at baseline (2014) and endline (2016) in the control and intervention arms, ITT and PP analyses adjusted for child’s age, child’s sex, and districts. CT cluster randomized controlled trial, Northern Togo, 2014–2016. CT, cash transfer; ITT, intention to treat; PP, per protocol.
(TIFF)

S3 Fig Mean HAZ ± SE of children aged 6–29 months at baseline (2014) and endline (2016) in the control arm and in the intervention arm, PP analysis adjusted for child’s age, child’s sex, and districts. CT cluster-randomized controlled trial, Northern Togo, 2014–2016. CT, cash transfer; HAZ, height-for-age z-score; PP, per protocol; SE, standard error.
(TIFF)

S1 Table Description of program components and modes of delivery. CT cluster-randomized controlled trial, Northern Togo, 2014–2016. CT, cash transfer.
(DOCX)

S2 Table Description of variables and indicators measuring the enabling environment. CT cluster-randomized controlled trial, Northern Togo, 2014–2016. CT, cash transfer.
(DOCX)

S1 Protocol Evaluation protocol for the Togolese CT program implemented in the Savanes and Kara regions, February 2014. CT, cash transfer.
(DOCX)

S1 CONSORT Checklist CONSORT checklist, CT cluster-randomized controlled trial, Northern Togo, 2014–2016. CT, cash transfer.
(DOC)

## Abbreviations

ASF: animal source food
BCC: behavior change communication
BW: birth weight
CCPW: community child protection worker
CHW: community health worker
CI: confidence interval
CT: cash transfer
DD: difference-in-differences
DDS: dietary diversity score
DDS7: DDS with 7 food groups
DHS: demographic and health survey
HAZ: height-for-age z-score
HBS: Household Budget Survey
HFIAS: Household Food Insecurity Access Scale
ICCM-Nut: integrated community case management of childhood illnesses and acute malnutrition
IFPRI: International Food Policy Research Institute
IPT: intermittent preventive treatment
IPV: intimate partner violence
ITT: intention to treat
IYCF: Infant and Young Child Feeding
LBW: low birth weight
MAD: Minimum Acceptable Diet
MDD: Minimum Dietary Diversity
MDD-W: MDD for Women
MMF: Minimum Meal Frequency
PP: per protocol
pp: percentage points
ROR: relative odds ratio
UCT: unconditional cash transfer
VAWI: Violence Against Women instrument
WDDS10: DDS for women with 10-food–group classification
