# Supplementary figures and images for: Evaluation of an unconditional cash transfer program targeting children’s first-1,000–days linear growth in rural Togo: A cluster-randomized controlled trial
Source: PLoS Med. 2020 Nov 17;17(11):e1003388. doi: 10.1371/journal.pmed.1003388 (PMC7671539; doi:10.1371/journal.pmed.1003388)

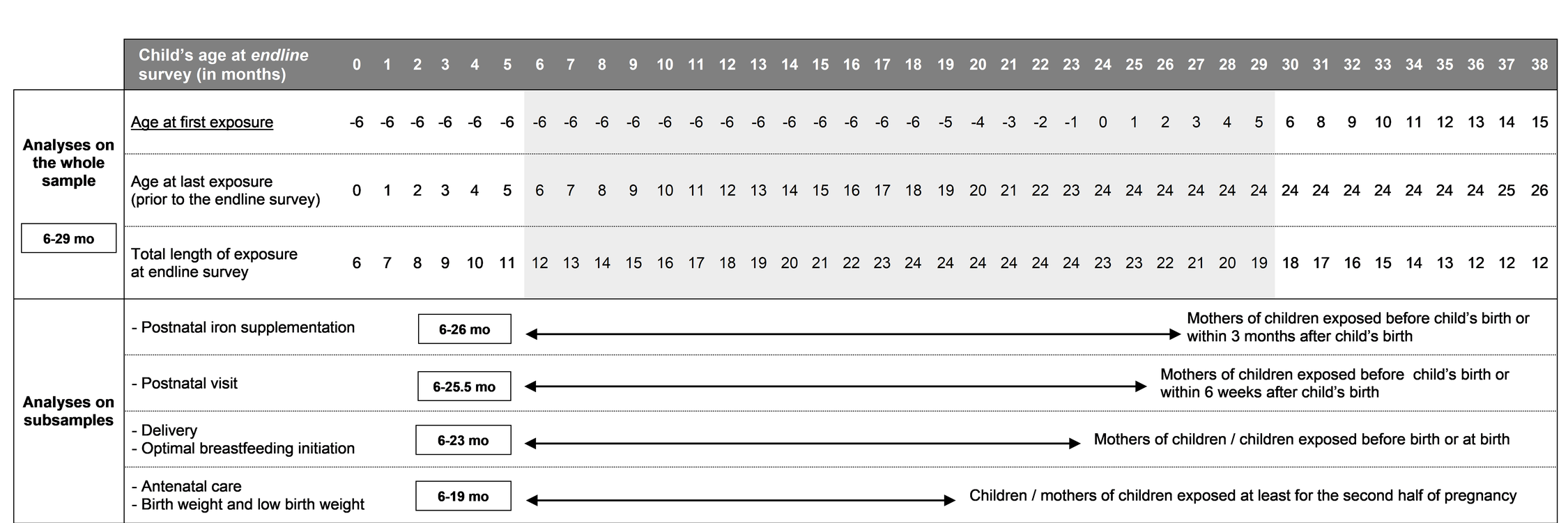

Supplement: S1 Fig — CT cluster-randomized controlled trial, Northern Togo, 2014–2016. CT, cash transfer. (TIFF) [file pmed.1003388.s001.tiff]
