# Supplementary material for: Evaluation of an unconditional cash transfer program targeting children’s first-1,000–days linear growth in rural Togo: A cluster-randomized controlled trial
Source: PLoS Med. 2020 Nov 17;17(11):e1003388. doi: 10.1371/journal.pmed.1003388 (PMC7671539; doi:10.1371/journal.pmed.1003388)
Supplement: S1 Table — CT cluster-randomized controlled trial, Northern Togo, 2014–2016. CT, cash transfer. (DOCX) [file pmed.1003388.s004.docx]

| Program components | |  | Mode of delivery | Beneficiaries | Organization in charge |
| --- | --- | --- | --- | --- | --- |
| **Cash transfers** - *implemented since 2014* | |  |  |  |  |
| - Monthly cash transfers: 5000 XOF/month (8.40 USD/month) - Bonus for women assiduous to sensitization meetings   To qualify for this reward, women were required to attend at least m+1 sensitization meetings, where m is the mean number of times women of a given village took part in sensitization meetings | |  | Delivered by postal agents   - In cash - To women - At payment points* directly in villages upon presentation of their beneficiary cards   **Women should not have to walk more than 5 km to reach the payment point* | - Pregnant women of at least 3 months - Mother of children aged 0-23 months - Mother of children aged 24-59 months suffering from acute malnutrition | - Ministry of the Post - Ministry of Grassroots Development - Technical support: World Bank |
| **BCC activities concerning children‘s rights and protection** - *implemented since 2014* | | | |  |  |
| - Birth registration - The Right to education - Physical violence towards children - Violence and sexual abuse towards children - Child labor - Child trafficking - Harmful traditional practices (such as honor killings, breast ironing) | - Early marriage - Female Genital Mutilation - Infanticide - Stigmatization of children accused of witchcraft - Fostering children in convents |  | Delivered by community child protection workers through:   - **Sensitization meetings**, organized monthly - **Home visits**, conducted as regularly as possible   Equipped with:   - An instruction manual - **Counseling cards** promoting children’s protection, with graphics and key counseling messages on the reverse side, to be used in BCC activities - **A follow-up booklet of households** | - All villagers, particularly women and mothers of young children | - Ministry of Social Action - Ministry of Grassroots Development - Technical support: World Bank |
| **ICCM-NUT Program** - *implemented since 2011* | |  |  |  |  |
| **BCC activities concerning health and nutrition-related issues**  *Focus on 12 essential family practices* | |  | Delivered by community health workers through:   - **Sensitization meetings,** organized monthly - **Home visits,** conducted as regularly as possible   Equipped with:   - **A manual** - **Counseling cards**, with graphics promoting health and key counseling messages on the reverse side, to be used in BCC activities - **A follow-up booklet of households** | - All villagers, particularly women and mothers of young children | - Ministry of Health - Technical support: UNICEF |
| - Maternal breastfeeding - Infant and young child feeding practices - Hand washing with soap - Utilization of Oral Rehydration Therapy and zinc in case of diarrhea - Utilization of impregnated bed nets - Vaccinations and vitamin A supplementation | - Uptake of health facilities - Pregnancy and antenatal care visits - Delivery and postnatal visits - Preventing of mother-to-child transmission of HIV - Hygiene personal practices - Water, sanitation and hygiene - Birth registration |  |  |  |  |
| **Screening and treatment of 3 common childhood illnesses + acute malnutrition**   - Malaria - Diarrhea - Acute respiratory infections - Acute malnutrition | |  | Delivered by community health workers whenever necessary  Equipped with:   - **A flow diagram listing danger signs** - Danger signs: children are referred to a health facility.   *Danger signs include*: cough for 14 days or more, diarrhea for 14 days or more, blood in stool, fever for the last 7 days or more, swelling of both feet, MUAC <115 mm + appetite loss (-> refer to nutritional rehabilitation center)   - No danger signs: children are treated at home by community health workers - **A medical kit** - Rapid diagnostic tests and antimalarial drugs to test and treat malaria - Oral rehydration salts and zinc tablets to treat diarrhea - Antibiotics to treat acute respiratory infections - Mid-upper arm circumference strip and ready to use therapeutic food to treat severe acute malnutrition | - All children aged 0-59 months | - Ministry of Health - Technical support: UNICEF |

S1 Table - Description of program components and modes of delivery - Cash transfer cluster randomized controlled trial, Northern Togo, 2014-2016
