# Supplementary material for: Evaluation of an unconditional cash transfer program targeting children’s first-1,000–days linear growth in rural Togo: A cluster-randomized controlled trial
Source: PLoS Med. 2020 Nov 17;17(11):e1003388. doi: 10.1371/journal.pmed.1003388 (PMC7671539; doi:10.1371/journal.pmed.1003388)
Supplement: S2 Table — CT cluster-randomized controlled trial, Northern Togo, 2014–2016. CT, cash transfer. (DOCX) [file pmed.1003388.s005.docx]

***S2 Table - Description of variables and indicators measuring the enabling environment. Cash transfer cluster randomized controlled trial, Northern Togo, 2014-2016.***

|  | **Variables, indicators, scores** |  | **Description** |
| --- | --- | --- | --- |
| WOMEN KNOWLEDGE | Global knowledge |  | Proportion of women with poor (0-8 pts), medium (9-12 pts), good (13-25 pts) global knowledge  *Sum of women’s knowledge on breastfeeding, nutrition, child’s health, pregnancy and delivery, hygiene, and birth registration (details below)* |
|  | Knowledge on breastfeeding |  | Proportion of women with poor (0-3 pts), average (4 pts), good (5-6 pts) knowledge on nutrition  *The mother knew: that the colostrum should be given to the child right after birth (0-1pt); that six months is the minimum age to introduce liquids other than breastmilk (0-1pt); that a child should be breastfed at minimum until 24 months (0-1 pt); that she should continue to breastfeed his child even if she is sick (0-1 pt); good practices to have enough milk (0-2 pts)* |
|  | Knowledge on nutrition |  | Proportion of women with poor (0-2 pts), average (3-4 pts), good (5-8 pts) knowledge on nutrition  *The mother knew: the timing of introduction of complementary foods (0-1pt); at least one vitamin A - rich food (0-1pt); how to feed a child after illness (0-2 pts); how to encourage a child to eat (0-2 pts); what to do when a child loses weight (0-2 pts)* |
|  | Knowledge on child’s health |  | Proportion of women with poor (0-1 pts), average (2-3 pts), good (4-7 pts) knowledge on health  *The mother knew: the timing of the first vaccination (0-1pt); the number of vaccination sessions a child should attend before his first birthday (0-1pt); from what age a child should be supplemented with vitamin A (0-1pt); how to prevent malaria (0-2 pts); what to do in case of childhood diarrhea (0-2 pts)* |
|  | Knowledge on pregnancy and delivery |  | Proportion of women with poor (0-2 pts), average (3 pts), good (4-5 pts) knowledge on health  *The mother knew: that she should attend at least 4 prenatal visits (0-1pt); that the best place to give birth is a health center (0-1pt); why it is important to give birth in health center (0-3 pts)* |
|  | Knowledge on hygiene |  | Proportion of women with poor (0-3 pts), average (4-5 pts), good (6-10 pts) knowledge on hygiene  *The mother knew: when she should wash her hands or those of her child (0-4pt); good practices regarding food hygiene, i.e. food preparation & storage (0-4 pts); how to ensure the safe disposal of human feces (0-2pt)* |
|  | Knowledge on birth registration |  | Proportion of women with poor (0pt), average (1 pt), good (2-3 pts) knowledge on hygiene  *The mother knew:*  *the legal deadline to register her child’s birth (45 days) (0-1pt); why it is important to register her child’s birth (0-2 pts)* |
| WOMEN EMPOWERMENT | ***Intra-household decision making*** | | |
|  | Women sole decision |  | Proportion of women with low (0-1 pts), moderate (2-3 pts), high (3-12 pts) decision making power  *1 point per item when the woman decided only by herself*  *Twelve items - Who decide: of the food to purchase to feed children; to bring a child to medical follow-up; to seek treatment if a child is sick; to enroll children in school; of the children's education/discipline; if she can use health care services for her own health; to use family planning; to have another child; whether she can work to earn money; how to use her own money; if she can go shopping outside the village; if she can visit her friends/family or travel away.* |
|  | ***Intimate partner violence (IPV) – partnered women only*** | | |
|  | Controlling behavior |  | Proportion of women who experienced at least once controlling behavior from their partner the last 12 months  *Partner's controlling behaviors****:*** *he insisted on knowing where she was at all times; he got angry if she spoke with another man; he was often suspicious that she was unfaithful; he tried to keep her from seeing her friends; he tried to restrict contact with her family of birth* |
|  | Emotional violence |  | Proportion of women who experienced at least once emotional violence from their partner the last 12 months  *Partner's emotional violence: he ignored her and treated her indifferently; he insulted her or made her feel*  *bad about herself; he belittled or humiliated her in front of other people; he did things to scare or intimidate her* |
|  | Physical violence |  | Proportion of women who experienced at least once physical violence from their partner the last 12 months  *Partner's physical violence: he slapped her, pulled her hair, he threw something at her that could hurt her* |
| EXPENDITURES | ***Household monthly per capita expenditures in CFA*** | | |
|  | Total monthly expenditures |  | Food expenditure + non-food expenditure |
|  | Monthly food expenditures |  | Monetary value of self-consumption + expenditure on markets |
|  | Monthly non-food expenditures |  | Rent, bills for water (excluding drinking water), electricity, combustible, phone, transport, health, schooling, clothing, purchase of assets, leisure, tobacco, other expenses. |
|  | Monthly ASF expenditures |  | Monetary value of self- consumption and purchase of : eggs, dairy products, flesh food |
