# Supplementary material for: Evaluation of an unconditional cash transfer program targeting children’s first-1,000–days linear growth in rural Togo: A cluster-randomized controlled trial
Source: PLoS Med. 2020 Nov 17;17(11):e1003388. doi: 10.1371/journal.pmed.1003388 (PMC7671539; doi:10.1371/journal.pmed.1003388)
Supplement: S1 Protocol — CT, cash transfer. (DOCX) [file pmed.1003388.s006.docx]

**EVALUATION PROTOCOL FOR THE CASH TRANSFER PROGRAM**

**IN THE SAVANES AND KARA REGIONS OF TOGO**

**February 2014**

**French National Research Institute for Sustainable Development**

**(*Institut de Recherche pour le Développement* [IRD])**

Montpellier, France

Dakar, Senegal

With scientific support from the International Food Policy Research Institute (IFPRI)

Washington, D.C., USA

Dakar, Senegal

**Table of Contents**

[I. Introduction 1](#_Toc50030064)

[II. The cash transfer project 2](#_Toc50030065)

[2.1. Objectives and standards 2](#_Toc50030066)

[2.2. Intervention areas 4](#_Toc50030067)

[2.3. Terms of transfers 4](#_Toc50030068)

[2.3.1. Targeting beneficiaries 4](#_Toc50030069)

[2.3.2. Transfer values 5](#_Toc50030070)

[2.3.3. Conditionalities 5](#_Toc50030071)

[2.4. Mechanisms of action 6](#_Toc50030072)

[III. Evaluation of the cash transfer program 7](#_Toc50030073)

[3.1. Specific evaluation objectives 7](#_Toc50030074)

[3.2. Methodology 7](#_Toc50030075)

[3.2.1. Approach 7](#_Toc50030076)

[3.2.2. Sampling 10](#_Toc50030077)

[3.2.3 Sample size 10](#_Toc50030078)

[3.2.4. Survey organization 11](#_Toc50030079)

[3.2.5. Data collection and indicators 12](#_Toc50030080)

[IV. Ethics 19](#_Toc50030081)

[V. Calendar 20](#_Toc50030082)

[VI. Partnerships 21](#_Toc50030083)

**List of acronyms**

CCPW Community Child Protection Workers

CDP Community Development Project

CHW Community Health Worker

CRENAS Centres de Réhabilitation Nutritionnelle Ambulatoire pour les Malnutris Aigus Sévères [Outpatient Nutritional Recovery and Education Centers]

CRENI Centres de Réhabilitation Nutritionnelle Intensif [Inpatient Nutritional Recovery and Education Centers]

CT Cash Transfer

CTC Community Therapeutic Care

EU European Union

HAZ Height-for-Age Z-score

IFPRI International Food Policy Research Institute

IRD Institut de Recherche pour le Développement [French National Research Institute for Sustainable Development]

MASSN Ministère de l’Action Sociale et de la Solidarité Nationale [Minister of Social Action and National Solidarity]

MDBAJEJ Ministère du Développement à la Base, de l’Artisanat, de la Jeunesse et de l’Emploi des Jeunes [Minister of Grassroots Development, Crafts, Youth, and Youth Employment]

ODK Open Data Kit

PHND Poverty, Health, and Nutrition Division

PPCM Programme de prise en charge de la Malnutrition [Integrated Community Case Management of Childhood Illnesses and Undernutrition (ICCM-Nut)]

SC School Cafeteria

SSN Social Safety Net

UMR Unité Mixte de Recherche [Joint Research Unit]

UNICEF United Nations Children's Fund

USP Unité de Soins Périphérique [Peripheral Care Unit]

WHZ Weight-for-Height Z-score

# **Introduction**

The Community Development Project financed by the World Bank in Togo is implementing an approach to social welfare via two social safety net (SSN) programs, namely: (a) school meals for approximately 38,000 primary school students in the country’s most vulnerable areas, and (b) labor-intensive public works employing 25,000 young people in rural areas. In light of these programs’ successes, and in an attempt to sustain Togo’s ongoing efforts to realize the establishment of social welfare policies that address the needs of the most vulnerable populations, the World Bank and the Togolese government opted to renew funding for the PDC, while adding an element dedicated to a conditional cash transfer pilot project (PDCPlus). This pilot project, in partnership with the Togolese government (MASSN and MDBAJEJ) and UNICEF, will launch in June 2014 in five districts in the Kara and Savanes regions and will last for 30 months. It will coordinate with a childhood malnutrition healthcare program carried out by regional health services in these districts and financed by UNICEF, and will incorporate certain flexible conditionalities (encouraged, not mandatory). The project is aimed at pregnant women and mothers of children under 24 months old; its objectives are to encourage behavior conducive to the protection and well-being of beneficiary children, and to improve their nutrition and health.

The current protocol concerns the impact evaluation of the cash transfer pilot project. It attempts to determine the effectiveness of cash transfers in conjunction with the program dedicated to preventing and treating acute malnutrition through changes observed in the following indicators: nutritional status, diet, child welfare, care, and morbidity; the protocol also aims to assess the program’s impact pathways. This evaluation will be conducted by the Joint Research Unit Nutripass of IRD, in scientific partnership with the PHND (Poverty, Health, and Nutrition Division) of IFPRI.

A process evaluation and program cost analysis are also planned, but these are addressed in other protocol documents.

# **II. The cash transfer project**

## **2.1. Objectives and standards**

The principal objective of the cash transfer (CT) project in Togo is to improve impoverished communities’ access to social safety nets. This project will also enable both testing the implementation of a cash transfer program and evaluating how the government might employ such a tool in the long term.

In an effort to improve the expected outcomes of this cash transfer project, a decision was made to test it in coordination with another community-based project, whose functioning already involves state services. This coordination should allow for the completion of a package of existing services to the benefit of the target group. From the public actions matching this description, the Integrated Community Case Management of Childhood Illnesses and Undernutrition program (hereinafter abbreviated as “ICCM-Nut”), implemented by the Ministry of Health and financed by UNICEF and the EU, was chosen. The ICCM-Nut is carried out by the Nutrition Service (Ministry of Health) in the Kara region (365 villages) and the Savanes region (200 villages). The ICCM-Nut’s objectives are to prevent malnutrition among children 59 months old and younger, and to provide treatment for severely malnourished children.

UNICEF provides personnel training and resources essential to treating severely malnourished children at Inpatient Nutritional Recovery and Education Centers (hereinafter abbreviated as “CRENI”) and Outpatient Nutritional Recovery and Education Centers (“CRENAS”), as well as necessary equipment and medicine. At the village level, the system established consists of a community health worker (CHW), who is assisted by a support committee often made up of volunteer mothers. The CHW organizes monthly meetings to track the growth of children up to 59 months old. When a child shows mild signs of malnutrition, they are placed into a group of “vulnerable infants”, who benefit from closer monitoring and dietary counseling. Severely malnourished infants who present no medical complications are cared for by CHWs at the community level, in order to avoid prolonged stays away from home at CRENAS facilities. However, any child exhibiting signs of severe malnutrition and experiencing medical complications is referred to the closest CRENI facility, in order to undergo a “nutritional recovery” fortified by medical and dietary treatment.

Hopefully, a synergy will develop between the ICCM-Nut and the cash transfer project, which would permit:

***-Promoting behaviors conducive to children’s welfare and well-being:***

The CT project has established some flexible conditionalities to encourage essential positive behaviors that promote the welfare and well-being of children. These behaviors include: obtaining a birth certificate, sending siblings to school when they are of age, keeping children living at home (as opposed to foster care), and pregnant women and mothers’ attendance at the monthly growth-tracking meetings. Educational gatherings centered around these themes and childrens’ rights will be organized. This complementary relationship between welfare and health/nutrition creates a “knowledge package”, whose purpose is to encourage mothers to invest in their children’s well-being and futures.

***-Improving the nutritional status of beneficiary children:***

The CT project will strongly encourage mothers’ participation in educational meetings regarding nutrition and hygiene, organized regularly by CHWs. As a result of mothers’ slightly increased purchasing power (via cash transfers), it is expected that they will be better able to implement the guidance they receive and to devote more resources to buying essential food items, thus providing a balanced diet for their children.

***-Better care for severely malnourished children:***

Community child protection workers (CCPWs) will conduct regular home visits, during which they will enlist and encourage mothers and guardians to attend children’s growth-tracking meetings; this regular and increased attendance will greatly aid CHWs in monitoring children closely and early detection of severe malnutrition necessitating medical care. Including these especially vulnerable children as CT beneficiaries will facilitate more comprehensive care for malnourished children and limit potential relapses.

## **2.2. Intervention areas**

The CT project will be concentrated in the two northern regions (Kara and Savanes) and some of the villages under ICCM-Nut supervision. The ICCM-Nut is active in 365 villages in the Kara region and 200 in the Savanes region. These localities, called CTC (“Community Therapeutic Care”) villages, were identified by UNICEF based on their isolation and distance from the closest peripheral care unit (UPS). Because the available financial resources will not allow for all CTC villages to be covered, the following refined targeting process will be used:

-Choosing the poorest districts, with the most concerning nutritional indicators, in both regions. The chosen districts are as follows: (a) Kpendjal and Oti from the Savanes region, and (b) Dankpen, Doufelgou, and Kéran from the Kara region.

-Using random selection to identify the villages to be covered within these districts.

The localities chosen for the pilot phase are very poor, remote villages, with little to no access to basic social services (for example, the closest healthcare unit is typically at least five kilometers away).

## **2.3. Terms of transfers**

### **2.3.1. Targeting beneficiaries**

Taking into consideration the extreme levels of poverty in targeted villages and the insignificance of the inequality between residents therein, a universal targeting method based on children’s ages made it possible to reach those most at risk for malnutrition. Additionally, the impact of pregnant mothers’ nutrition on the healthy development of their pregnancies and their children was taken into consideration, and led to the inclusion of all women pregnant for at least three months in the beneficiary group. Community members deemed this inclusion method to be extensive and equitable, and judged it would preserve villages’ social cohesion. Thus, in each targeted village, cash transfer recipients will be women who have been pregnant for at least three months and the mothers and guardians of all children up to 24 months old. This reflects the concept of “1,000 days”, which is considered worldwide to be a window of opportunity for intervention and combating undernourishment. Furthermore, any mother or guardian of a child under five years old and suffering from severe acute malnutrition (with or without medical complications) will be systematically included. In polygamous households, each mother of an eligible child will benefit from cash transfers through the eligibility of said child. In cases where two children (not twins) with the same mother both qualify as beneficiaries, only the eligibility of the youngest child will be taken into consideration. In cases of the death of an eligible mother, the child’s caretaker (as chosen by the family) will be registered in her place. A miscarriage or the death of an eligible child voids a mother’s eligibility.

The following exceptions, however, shall be granted:

- A mother of twins 24 months old or younger will receive two cash transfers;
- A mother who, in addition to her own eligible child, is the guardian of an eligible child whose mother is deceased shall be considered equivalent to a mother of twins.

### **2.3.2. Transfer values**

The monthly disbursement is 5,000 CFA francs per eligible child, and is paid for a minimum of 12 months and a maximum of 30 months. This decision will permit the project, over the course of at least three years, to reach a significant number of beneficiaries (an estimated 8,000 children are eligible) while testing the functionality of the system in place.

### **2.3.3. Conditionalities**

The selected conditionalities will be flexible and non-exclusive, and should help communities adopt behaviors conducive to the well-being and welfare of children. Mothers who meet these conditionalities will receive a small bonus at the end of the program. The conditionalities for each child are as follows:

- Possession of a birth certificate or an attestation to the ongoing procuration of a civil

status document

- Mother/guardian’s attendance at educational meetings concerning childhood needs

- Mother/guardian’s regular attendance at monthly childhood growth-tracking meetings

- At least four antenatal consultations (for pregnant mothers)

- Keeping children under 15 years old living at home (as opposed to in foster care)

- Primary school attendance for all children of age in the household, in order to avoid

child trafficking and exploitation

Community agents in charge of the project’s implementation will provide each household with the necessary accompaniment (information, referrals, administrative procedures, etc.) for the purpose of promoting the adoption of these proposed conditionalities.

## **2.4. Mechanisms of action**

There are multiple ways in which this program may act upon childhood well-being and development. Leroy et al. recently described the pathways by which cash transfers, conditional or not, can theoretically improve children’s nutritional status, by developing a model of causal pathways. This model has been adapted for the current project in order to identify how the latter might influence children’s diet, health, and nutritional status.


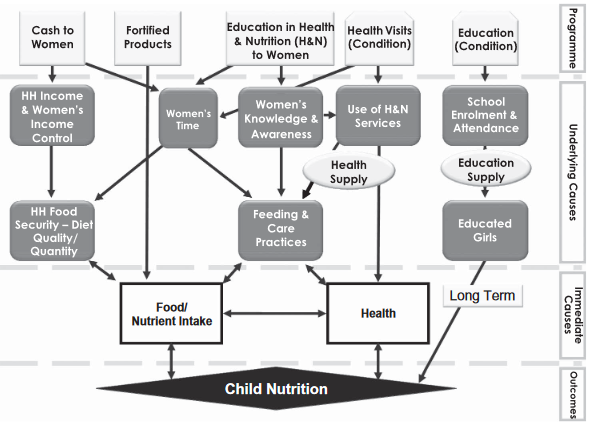


Figure 1 : Mechanisms of conditional cash transfers’ potential influence on nutritional status (from Leroy et al., 2009^[[1]](#footnote-1)^)

# **Evaluation of the cash transfer program**

## **3.1. Specific evaluation objectives**

The objective is to evaluate the impact of cash transfers in conjunction with the ICCM-Nut, through documenting and analyzing the changes in several indicators relevant to children: diet, nutritional status, morbidity, and child welfare. This objective will also include an analysis of beneficiary households’ use of funds received and an analysis of the project’s impact pathways.

## **3.2. Methodology**

### **3.2.1. Approach**

The framework for analyzing the program’s impact will be experimental: all villages in the study area (*n*=162) will be randomly assigned either to an intervention group or a control group. Over a period of 24 months, cross-sectional surveys of children aged six to 29 months and their mothers will be conducted in these villages; one survey will take place before the program begins (hereinafter referred to as “baseline”) and the second will be conducted at the program’s end (hereinafter referred to as “endline”). A difference-in-differences analysis will permit both to determine whether situational evolution between the baseline survey and the endline survey differs significantly between the intervention group and the control group, and to conclude what impact is attributable to cash transfers and/or the flexible conditions with which they are associated.

Rationale for selected impact evaluation design:

1. ***Repeated cross-sectional vs. longitudinal design*.** We propose to use a repeated cross-sectional survey design rather than a longitudinal design primarily because our previous experience has shown that usual delays in program roll-out and full implementation can have profound effects on the evaluation study cohort (e.g. children aging out of the “window of opportunity” for maximum nutritional impact of the intervention – see point 2 below), making the cohort unusable for assessing impact on age-sensitive outcomes such as child feeding practices or stunting. Additional constraints of longitudinal designs include the logistical complexity and related additional cost of tracking individual children over time, compared to sampling children of a given age in a repeated cross-sectional design. However, given that our team has extensive experience with both types of designs, we are open to discussions with UNICEF and the World Bank regarding alternative designs.
2. ***Age range of children included in evaluation samples.*** The assessment of the impact of nutritional interventions on child anthropometry (as well as other outcomes) should consider the age at which assessments should be made to detect the greatest difference between intervention and comparison areas. Evidence suggests that (1) the *longer* children are exposed to early nutrition inputs (within the first 1,000 days, or from conception to the child’s second birthday) and (2) the *earlier* they are exposed within this period, the greater the impact will be.

Furthermore, achieved height-for-age is likely to be greatest between intervention and control children in the age period *after* the peak age of growth faltering (i.e., after 18-24 months of age).

The logic behind investments made from conception until the child’s second birthday is that they are progressive and cumulative; hence our ability to detect a significant impact on anthropometry particularly, will be greatest among those children who were exposed to interventions in the entire period preceding the peak age of growth faltering. This means that the age at which impact on anthropometry should be assessed is dependent on (1) the child’s age at the onset of their exposure to program interventions, (2) the total duration of exposure to interventions within the 9 to 24 month target age-focus, and (3) the peak age of growth faltering.

As impact on intermediary outcomes such as complementary feeding indicators will also be measured, we have to make sure that the corresponding age group (i.e. children > 6 months of age) is well represented in our sample.

We are applying these principles to define the exact age group for whom to assess impact on feeding and nutritional outcomes, taking into account demographic and logistical considerations.

1. ***Duration between baseline and endline surveys*.** Although the proposed pilot program will last 30 months, the need to ensure that baseline and endline assessments are conducted at the same time of the year (and thus minimize the impact seasonality has on key indicators of interest) means the duration between baseline and endline assessments should be 24 months apart (allowing extra time for preparation and post-survey analysis).

Given the considerations outlined above, the duration of the program (30 months), and logistical considerations about sample sizes at particular age ranges, we propose sampling children six to 29 months of age at endline (two years after the baseline). Sampling the six-to-29-month age range is a compromise that takes into account a minimum program exposure of 12 months and first exposure to the program no later than six months of age. These criteria are based on the assumption that the main program intervention of cash transfers will begin immediately following the baseline survey and continue for 30 months, although at the time of the endline survey no child would have been exposed to the program for more than 24 months. Any delay in the start of cash transfers will have an impact on the age range of children to be sampled.

Table 1 below illustrates the age of first exposure, and duration of exposure of children of different ages, assuming a period of 24 months between baseline and endline surveys, and an immediate start to the cash transfer program after the baseline survey:

Table 1 : Program exposure of sample age range at endline

| **Age (mo)  at Endline** | **Age (mo) at First Exposure to Program** | **Age (mo)at Last Exposure to Program** | **Total Program Exposure (mo)** |
| --- | --- | --- | --- |
| 0 | -6 | 0 | 6 |
| 6 | -6 | 6 | 12 |
| 12 | -6 | 12 | 18 |
| 18 | -6 | 18 | 24 |
| 24 | 0 | 24 | 24 |
| 30 | 6 | 24 | 18 |
| 36 | 12 | 24 | 12 |
| 42 | 18 | 24 | 6 |
| 48 | 24 | 24 | 0 |
| 54 | N/A | N/A | 0 |
| 60 | N/A | N/A | 0 |

Extensive information will be collected at the levels of villages, households, mothers, and children, in order to:

**i) determine the program’s impact on several pertinent results**

- Diets of children and their mothers
- Care and welfare of children
- Morbidity
- Anthropometry of children

**ii) understand the ways in which the program works**

- Women’s use of funds received
- Tracking of household expenses
- Use of health services
- Women’s roles and decision-making power within the household
- Behavioral changes relative to questions around children’s nutrition, hygiene, health, and welfare
- Household food security

**iii) describe the analysis’s context and take into account potential confounding factors**

- Village characteristics (facilities, equipment, etc.)
- Household demographics and socioeconomic characteristics
- Jobs and education levels of heads of households and women
- Hygiene
- Economic shocks endured by households

### **3.2.2. Sampling**

The study will be of an experimental type, involving randomization at the village level. Five districts in the Savanes and Kara regions, representing a total of 273 villages, were initially selected using the criteria of poverty and malnutrition rates. Random distribution between the control group and the intervention group was stratified by district, and the village selection was proportional to each district’s population in order to reach a desired 4,800 children in the control group aged 24 months or younger. At the end of this process, 81 villages in total were placed in the intervention group. In order to balance the sampling at cluster level, 81 villages were selected from the remaining 192 and placed in the control group.

All pregnant women (from the third month of pregnancy onward) and children under 2 years old living in the selected villages will be enrolled in the program. The baseline and endline surveys will sample, respectively, children aged six to 59 months old and six to 30 months old who live in these 162 villages, as well as their mothers.

### **3.2.3 Sample size**

Sample size estimates are calculated based on changes in different impact indicators; HAZ and stunting and are based on the following principles:

- Using tests that compare means and prevalence between the two groups;
- Taking into account the design effect since randomization is carried out at the village level rather than individually;
- Using a conservative approach which consists of ignoring the fact that randomization is stratified by district;

The following parameters are fixed:

- 〈risk at 0.05;
- power at 90% ;
- 162 villages to be randomized (81 per group);
- Missing/invalid data at 10%;
- An intra-village correlation coefficient (ICC) that varies from 0.02 to 0.15

Based on the sample size estimates, we propose a sample size of 802 children aged six to 23 months/group, at baseline. This sample size will allow us to detect a difference of

9 points in prevalence of stunting and 0.20 in mean HAZ. This sample size will be extended to 1,020 children aged six to 29 months/group at baseline.

### **3.2.4. Survey organization**

Both the baseline and endline surveys will take place during home visits conducted by qualified interviewers, duly selected and trained for this specific study. They will be directly supervised and evaluated by experienced agents. The questionnaire will be administered confidentially, ideally over a period of 90 to 120 minutes, and will be followed by 10 to 15 minutes of anthropometric measuring conducted under standardized conditions. The questionnaires will be digital and loaded onto tablet PCs (via the Open Data Kit [ODK] system), except for the questionnaires regarding village characteristics and household composition.

Four interviewer-pathfinder duos will be charged with notifying and sensitizing the villages and households involved in the study. They will explain the study objectives, inform subjects of their next interviews, and obtain the necessary prior approval. These duos will also be in charge of completing the questionnaire concerning the village and will take a census of all members of households involved in the study to complete the household composition questionnaire. The duos will therefore pass from village to village a few days before the interviewers visit.

Approximately 30 interviewers will be recruited and trained on the questionnaires and anthropometric measurements. Five supervisors will be hired, each responsible for a team of six people. In order to guarantee the quality of the data, supervisors will initially accompany interviewers in person (thus making sure they are correctly administering the questionnaires, correctly entering collected data, etc.). A dedicated server will receive regular transmissions of data, so study directors can remotely monitor both the figures and work progress.

### **3.2.5. Data collection and indicators**

Data will be collected at different levels, namely, households, mothers, and children. The following is a list of the main components and key indicators to be gathered:

❖ **HOUSEHOLD LEVEL**

**Household composition and characteristics:** household size, head of household’s sex, revenue source, household members’ education levels, children’s schooling (past and present), children’s participation in household income generation, number of foster and/or adopted children in the household and reasons for their being there.

**Household socioeconomic level:** This will be evaluated by assessing housing quality and household assets. We are particularly interested in: the quality of building materials (roof, walls, floor), the presence of an electrical connection and running water, the presence or absence of a number of everyday household objects, productive assets, livestock, and agricultural land. A synthetic index representing households’ economic levels will be created using a factor analysis; this technique consists of weighting different categories of a number of variables which indirectly reflect household affluence. The resulting score is a dimensionless variable, interpreted for each household as an approximation of its level of wealth. The score could be used in analyses as a continuous variable, but after categorization into tertiles or quintiles, it may also be used to classify households by income level.

**Household food expenses**: These will be obtained through a 24-hour diet recall, either over a week or a month depending on whether food is purchased daily, weekly, or monthly. All of these expenses will be scaled to a monthly figure, totaled, and divided by the number of adult equivalents in the household. The number of adult equivalents is based on the value of individual energy needs with regard to age, sex, and level of physical activity, according to international values. The reference individual is a man between 30 and 59 years old, moderately physically active, who requires 2,450 Kcal daily. Thus, if an individual’s daily energy need is 1,225 Kcal (roughly equal to that of a nearly four-year-old girl), they are counted as 0.5 of an adult equivalent.

**Household non-food expenses:** These will be calculated based on the following items: lodging (rent, maintenance), cooking fuel source, electricity, water, health and education, hobbies, personal hygiene, clothing, shoes, vehicles, beauty care services, and communication. All of these expenses will be scaled to a monthly figure, which will then be weighted according to the household’s adult equivalent number as described above.

**Household food insecurity access scale (HFIAS):** The HFIAS scale permits a holistic look at households’ food insecurity, based on consequences and feelings stemming from recent experiences with food insecurity and strategies developed to deal with it. This instrument is inspired by the Radimer-Cornell scale, developed and employed in the United States within the framework of its federal food assistance programs, and has been adapted for use in developing countries following several tests and validation experiences. Scores are calculated based on responses to nine questions that address the various dimensions of food insecurity in order of increasing importance, according to current recommendations. Each response is marked between 0 (a “no” response) and 3 (1 to 3, depending on how frequently the household experiences the situation in question). The reference period comprises the prior month. The final score will thus range between 0 (indicating a food-secure household) and 27 (maximum food insecurity). Additionally, households’ experiences with the most severe consequences place them in one of four categories (food-secure and slightly, moderately, or severely food-insecure).

**Household economic shock:** Some households may experience economic shock during or just before the program that affects the family environment and, consequently, children. It is therefore invaluable to glean this information retrospectively in the context of this evaluation. If indeed a household experiences economic shock during the program, it may explain any possible lack of positive program impacts.

**Hygiene and sanitation:** Household hygiene can have a large impact on children’s health and nutritional status. We will employ spot checks to estimate the cleanliness of the property and its inhabitants, which have proved to be an efficient, non-intrusive, quick, and affordable approach. A short questionnaire on household sanitation (toilets, wastewater systems, and trash) will supplement this method.

❖ **PERTAINING TO MOTHERS**

**Mother’s characteristics:** This component consists of mothers’ civil statuses, education levels, and revenue sources.

**Schedule and child care:** Mothers’ schedules will be examined, as they can directly influence dietary practices and child care. The time mothers spend taking care of their children (meals, games, healthcare) will be observed with particular interest.

**Mother’s status, autonomy, and decision-making power:** This component will assess women’s household autonomy (particularly with regard to finances), the treatment they endure within the household (abuse, insults, anxieties, etc.), their involvement in sensitization meetings and social/community networks, and their decision-making power relative to issues of diet, health, education, family planning, and children’s rights within their households.

**Use of health services and attendance at informational sessions:** This component will take into account pregnancy history, household situations during the course of pregnancy (reduced work, improved diet, etc.), the number of pre- and post-natal consultations, birth setting and external help received, and mothers’ involvement in training sessions on children’s health, diet, and rights.

**Mother’s knowledge, attitudes, and practices (“CAP”):** The program aims to improve mothers’ knowledge bases in order to impact their dietary practices and their conduct relating to the caretaking, hygiene, nutrition, and rights of their children. After adjusting for certain variables such as education and socioeconomic levels, changes in the CAP between the baseline and endline surveys will be examined to evaluate the program’s impact pathways. The CAP questionnaires will be adapted to the content of the monthly information sessions planned in the project.

**DIetary diversity:** A dietary diversity score will be calculated for each mother following a qualitative recall of items consumed over the previous 24 hours. These scores will be determined by tallying the number of food groups consumed out of 12 total food group classifications. However, as there are currently no international recommendations for an optimal number of food groups or a “good dietary diversity” threshold for women of childbearing age, information will be collected from a larger number of groups and subgroups; thus, indicators can be constructed *a posteriori* according to recommendations adopted in the future (which should soon be implemented on an international level, hence during program implementation).

**Anthropometry:** Mothers’ height and weight will be measured in accordance with WHO recommendations. Along with body mass index, this raw data can be used to evaluate mothers’ nutritional statuses, which are known as a major factor in newborns’ nutritional statuses at delivery.

❖ **PERTAINING TO CHILDREN**

**Child’s health:** This component will gather information regarding possession of health records, children’s immunizations, growth charts, and children’s morbidity over the previous 15 days. Additionally, in cases of illness, mothers’ appetites and responses to said illnesses will be assessed during consultations at a health center (or elsewhere).

**Dietary practices:** Standard dietary practices indices for infants and young children, as recommended by the WHO, will be calculated following a qualitative recall of children’s feeding practices over the last 24 hours—for example, dietary diversity (number of food groups consumed out of seven total), frequency of ingestion (total number of meals and snacks consumed), consumption of foods of animal origin, consumption of iron-rich products, etc. Retrospective questions about breastfeeding will also be asked (initiation and cessation, exclusive breastfeeding), as well as questions about the introduction of complementary feeding and how the mother feeds her child (preparation of specially adapted meals, help with eating, encouraging consumption).

**Child welfare and rights:** The relevant MICS (Multiple Indicator Cluster Surveys) module will be used. Among other items, it includes questions about birth registration, child labor, schooling, child marriage, children’s education and discipline, and disabilities. It will be particularly important to observe whether attitudes and practices evolve with the program.

**Anthropometry:** Children’s height and weight will be measured in accordance with WHO recommendations in order to calculate height-for-age and weight-for-height nutritional indices, and will be expressed as *z*-scores using the WHO reference curves for growth from 2006.

❖ **PERTAINING TO SCHOOL-AGED SIBLINGS**

Even if there are no plans to specifically evaluate school cafeteria programs, it would be interesting to ask questions about children who benefit from them (namely those in primary school). We have therefore planned a minor module concerning the education of beneficiary children’s older siblings (attendance, grade level, assigned school). As school-aged children are expected to be in school during interviewers’ visits, the module will be posed directly to mothers.

### **3.2.6. Data analysis**

Statistical analyses will be performed using either R or STATA software. These analyses will take into account the characteristics of the sampling plan in strata (prefectures) and clusters (villages), and will be weighted according to village population sizes. The *p*-value, alpha, will be set at 0.05 and used as the threshold of significance for all comparisons.

Before the actual impact analysis, the results regarding indicators of interest will be presented by randomization group (intervention/beneficiary and control/non-beneficiary) at baseline in a descriptive fashion and by randomization group and prefecture at endline. Results concerning quantitative variables will be expressed in arithmetic mean values, while those dealing with qualitative variables will be expressed as frequencies (in percentages).

In order to evaluate program impact on the indicators of interest, the difference-in-differences method will be used. This method consists of comparing the intervention group to the control group both before and after the introduction of cash transfers. The underlying hypothesis is that absent the cash transfer program, the mean evolution of the indicators of interest should be the same in both groups. By considering an interaction term between the phase (baseline or endline) and the randomization group (intervention or control), this method permits evaluating whether the indicators’ evolution between the baseline and endline surveys differs significantly between the two groups. In other words, the difference-in-differences estimator compares the difference observed in the intervention group before and after program implementation with that observed in the control group. It also enables taking pre-existing differences between the intervention and control groups into account, as well as temporal effects, and thus allows for a conclusion on the existence of an impact attributable to cash transfers and/or the flexible conditionalities associated with them.

To this end, quantitative variable analysis will be conducted with the help of linear regression models, while qualitative binary variable analysis will be conducted with the help of logistic regression models. We will formulate
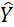

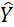
 (the estimation of the indicator of interest) and
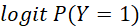

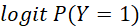
 depending on whether we are interested in, respectively, a quantitative variable or a qualitative binary variable, in the following form:

**
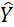
=
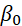
+
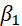
*phase+
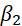
*group+
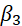
*phase*group**

Here, “phase” represents the survey (coded 0 for baseline and 1 for endline) and “group” represents the randomization group (coded 0 for the control group and 1 for the intervention group).
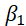
 is the effect of the phase (the difference between baseline and endline surveys),
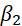
 represents the difference between individuals in the intervention group and the control group, and
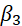
 represents the intervention’s effect on the intervention group or the difference-in-differences estimator.

**Using birthweight as an example, the table and figure below illustrate the significance of the various coefficients described above:**


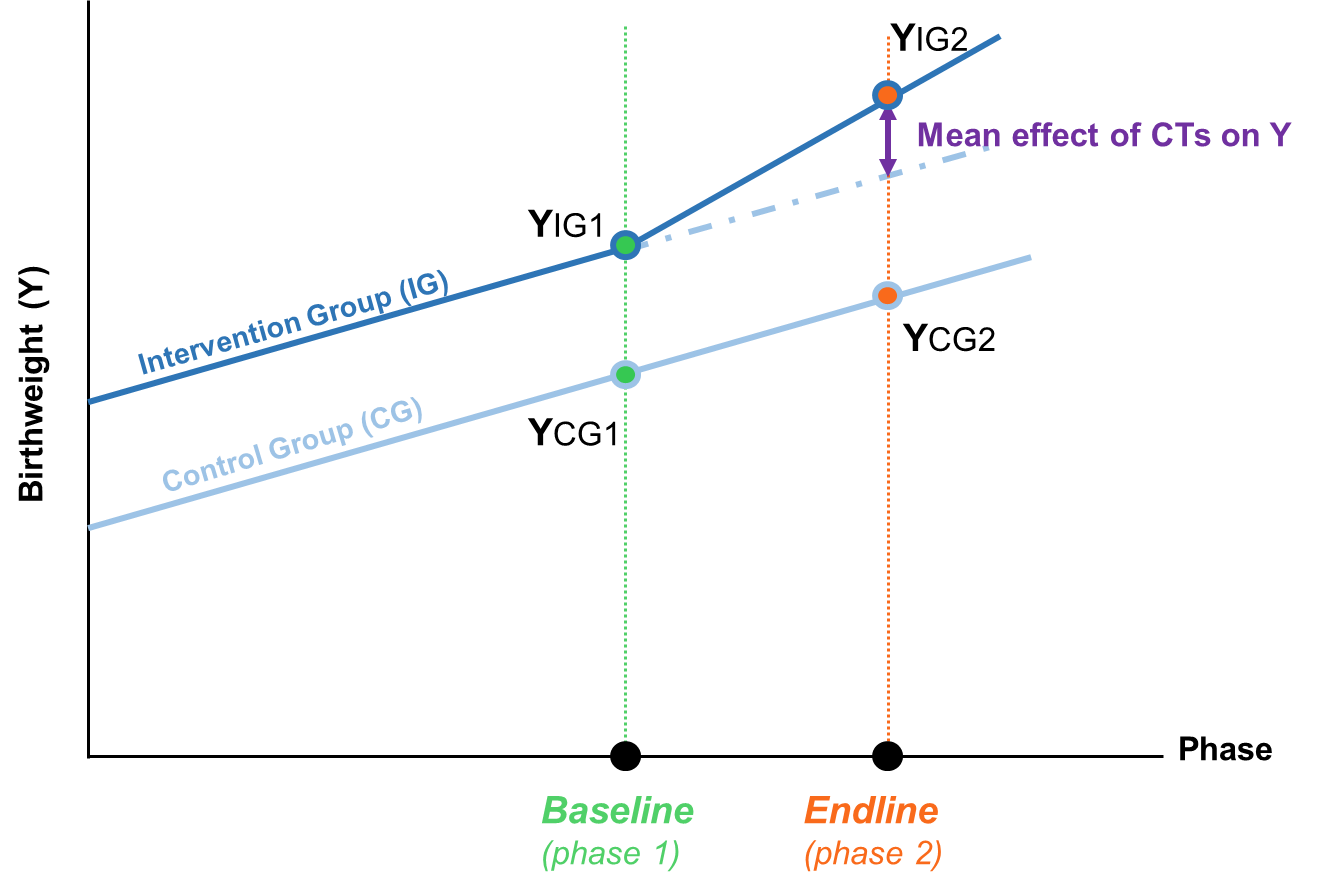


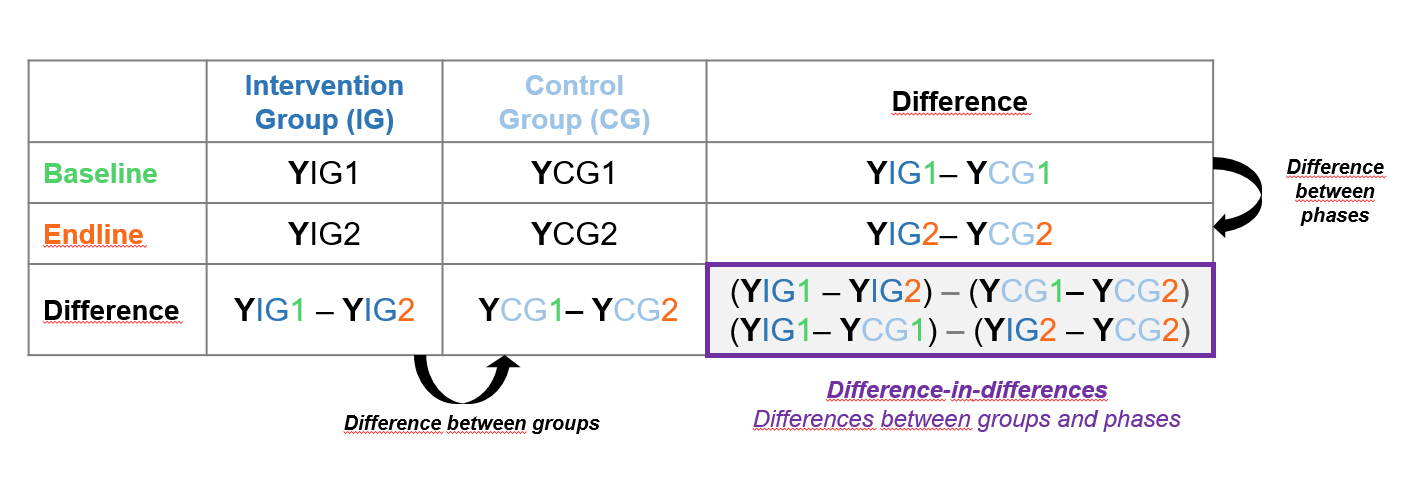


Initially, analyses will be carried out via intention-to-treat (ITT) analysis; mother-child pairs (“CME”) and households will be analyzed within their initial randomization group, whether or not they have actually received cash transfers. A later set of analyses will use per-protocol analysis (or treatment-on-the-treated [TOT]); only mother-child pairs who have actually received cash transfers will remain in the intervention group, and those pairs who have not will be excluded from further analyses.

Pre-specified outcomes to be analyzed are as follows:

Primary outcomes:

**Child’s anthropometrics**

- HAZ

Secondary outcomes:

- Stunting
- WHZ, wasting

Intermediate outcomes:

**Nutrition pathway**

- Child’s nutrition: IYCF practices (DDS7, MDD, MMF, MAD, iron-rich foods), consumption of animal source foods (ASF)
- Mother’s nutrition: MDD-W, DDS 10, number of meals, consumption of ASF
- Household food insecurity: HFIAS (HHS)
- Household food expenditures (as opposed to non-food expenditures)

**Health pathway**

- Child’s health: morbidity over the previous 15 days, child’s health since birth as perceived by the mother, vaccinations, vitamin A supplementation, deworming, regular medical follow-ups and age at last medical follow-up, health-seeking behavior (children taken to a health center if sick)
- Maternal health: antenatal and postnatal care (number of consultations, stage of pregnancy at first antenatal visit, malaria IPT, tetanus vaccines, iron supplementation) delivery in health facilities, delivery assisted by a skilled birth attendant, birth weight and LBW of newborns

**Hygiene pathway (through spot checks)**

- Child’s hygiene: cleanliness of hands, face, hair and clothes
- Mother’s hygiene: cleanliness of hands, face, hair and clothes
- Yard’s sanitary conditions: animal feces, waste

**Knowledge and awareness pathway**

- Mother’s knowledge of nutrition, health, and hygiene

**Women empowerment pathway**

- Decision-making power
- Intimate partner violence (IPV)

**Child welfare pathway**

- Child fostering
- Enrollment in school
- Birth registration

# **IV. Ethics**

Overall, the evaluation will conform to the ethical standards common to this kind of work; the present protocol will be submitted to the Togolese national ethics committee for approval.

The following standards will be applied:

-Information and request for authorization from community authorities: UNICEF and the CDP will be charged with alerting relevant authorities (mayor, prefect) to the establishment of this cash transfer program and its evaluation. In each study village, IRD teams will visit village authorities (village and religious leaders) in order to present the study’s objectives and implementation and obtain their authorization.

-Free and informed participation: each household or individual participating in one of the above activities will receive detailed written information about the study’s objectives and implementation, with regard to their participation in it. These elements will also be explained during initial sensitization, either in the respondent’s native tongue or a language they understand easily, and questions may be asked at this time if desired. Consent will be obtained via the head of household (or their spouse, if absent), though any members of the household reserve the right to opt out of the survey without any negative consequences for themselves, their families, or their loved ones. A household or individual may also revoke consent at any time, postpone interviewers’ visits, or refuse to answer any questionnaire items, all without needing any justification.

-Risks: the evaluation does not represent any risks to participants, as it consists simply of questionnaires administered by interviewers and some painless, non-invasive anthropometric measurements.

-Confidentiality and anonymity: the confidentiality of responses will be guaranteed at every step of the survey, from data gathering to the final analysis and publishing of results; questionnaires will be processed anonymously and no computerized results will contain any identifying information.

-Expected benefits: these primarily concern participating mothers and children, as cash transfers will hopefully better their everyday lives (through improved nutritional intake, better medical monitoring, etc.) and lead to a reduction in morbidity and an enhanced nutritional status. The benefits will also concern other children in the household via flexible conditionalities (schooling of eligible children’s siblings, avoiding foster care); additionally, these children should profit from their mothers’ knowledge of nutrition, health, and child welfare, accrued through monthly sensitization sessions. The entire household could potentially benefit from the fact that the cash may be freely spent; notably, household food security is expected to improve. Finally, benefits will also pertain to the entire local community, as the study should lead to improvements in the interventions themselves or those of the same type; in a broader sense, the study will allow national and international decision-makers to be better informed about the potential effects, whether beneficial or detrimental, of this program.

-Comparison group: program effects will be evaluated through a comparison with a group of women (and their children) who will not receive cash transfers, but who nonetheless will benefit from UNICEF activities comprising detection and treatment of severe acute malnutrition.

-Emergencies: all participants, whether from the beneficiary group or the control group, are eligible for medical treatment in the event of emergencies observed during visits; medical costs will be covered in cases of severe health problems, as treatment of malnutrition is already provided for as part of the ICCM-Nut.

# **V. Calendar**

A calendar detailing the preparation and execution of the initial survey is included below. Currently, the survey is scheduled to begin in May 2014 and is projected to last about 45 days.

The final survey will take place 24 months after the first, in May 2016.

# **VI. Partnerships**

The entire evaluation will unfold in close collaboration with the organizations and institutions involved in the project, beginning with UNICEF, the World Bank, and the CDP (which depends on the Ministry of Grassroots Development, Crafts, Youth, and Youth Employment, as well as the Regional Department of Social Action (“DRAS”). On the other hand, a committee dedicated to the monitoring and orientation of project impact studies will be set up and composed of a representative from each of the following entities: the Ministry of Tutelles, UNICEF, the Ministry of Social Action and National Solidarity, the Ministry of Health, the national general directorate of statistics and accounting, the technical secretariat of community development projects and social safety nets, and the World Bank. This committee will be set up very soon through a decree issued by the Ministry of Grassroots Development, Crafts, Youth, and Youth Employment.

1. Jef L. Leroy, Marie Ruel & Ellen Verhofstadt (2009) The impact of conditional cash transfer programmes on child nutrition: a review of evidence using a programme theory framework, Journal of Development Effectiveness, 1:2, 103-129, DOI: 10.1080/19439340902924043) [↑](#footnote-ref-1)
